# Gas chromatography-time-of-flight mass spectrometry (GC-TOFMS)-based metabonomic response of *Salvia miltiorrhiza* flowers to cadmium stress

**DOI:** 10.7717/peerj.21149

**Published:** 2026-04-27

**Authors:** Jun Yuan, Jianling Wang, Rongpeng Liu, Haihui Fu, Xiaoyun Wang

**Affiliations:** 1 School of Traditional Chinese Medicine/School of Life Sciences, Jiangxi University of Chinese Medicine, Nanchang, China; 2 Jiangxi Key Laboratory for Sustainable Utilization of Chinese Material Medical Resources, Jiangxi University of Chinese Medicine, Nanchang, China; 3 School of Pharmacy, Jiangxi University of Chinese Medicine, Nanchang, China; 4 Key Laboratory of Crop Physiology, Ecology and Genetic Breeding, Ministry of Education, Jiangxi Agricultural University, Nanchang, China

**Keywords:** Flowers of SM, Cd stress, Amino acids, Sugars, Organic acids

## Abstract

Cadmium (Cd) poses a threat to plants and humans. As a commonly used Chinese medicinal material, *Salvia miltiorrhiza* (SM) roots have been investigated in many studies, but few studies have investigated SM flowers. In particular, there is a lack of research on the response of SM flowers to Cd stress. Here, the metabolomic mechanisms underlying the response of SM flowers to Cd stress were analyzed. Six kinds of metabolites were detected: amino acids, organic acids, sugars, lipids, alcohols, and others. Among them, organic acids accounted for the largest proportion, followed by sugars and amino acids. Principal component analysis (PCA) and orthogonal partial least squares discriminant analysis (OPLS-DA) clearly differentiated the control from the Cd-treated groups. A total of 31, 40, and 49 differential metabolites (DMs) were detected in 25 (HT1), 50 (HT2), and 100 (HT3) mg·kg^−1^ Cd-treated groups, respectively. These DMs were mainly enriched in alanine, aspartate and glutamate metabolism, glyoxylate and dicarboxylate metabolism, butanoate metabolism, citrate cycle (TCA cycle), and glycine, serine and threonine metabolism. Among the metabolites, oxamic acid and citric acid contributed most to the down-regulated differential amino acids and differential organic acids, respectively; xylose and 1-kestose contributed most to the up-regulated differential sugars; tryptophan and arachidic acid contributed most to the up-regulated amino acids and organic acids, respectively. These findings suggested that SM flowers might resist Cd stress through restructuring the cell-wall framework, modulating membrane fluidity, and regulating intracellular soluble components. These findings provide new avenues for exploring how medicinal plant resources cope with heavy-metal stress.

## Introduction

Heavy metals accumulate in soils through both natural (*e.g*., rock weathering, atmospheric deposition) and anthropogenic sources (*e.g*., sewage irrigation, excessive use of fertilizers and pesticides) (Zhang et al., 2021b; Ahmed et al., 2022; Zhou et al., 2022). This seriously affects plant survival and, in turn, threatens human health through the food chain (Popovic, Djordjevic & Polic, 2001). Over 16% of the surveyed soil samples in China are contaminated, and cadmium (Cd) is the primary soil pollutant in China (Ministry of Environmental Protection of the People’s Republic of China, 2014; Ahmed et al., 2022). As a non-essential element in biology, Cd is easily absorbed by plant roots, and transported to the aboveground parts of the plant for accumulation (Yuan, Xue & Han, 2021). With high biological mobility, non-degradability, and ease of accumulation, Cd seriously threatens the global environment and ultimately endangers human health (Dubey et al., 2014; Zhao et al., 2022). Therefore, it is crucial to understand how plants respond to Cd stress in order to enhance their tolerance and regulation (Wang, 2023).

Under Cd stress, plants undergo stress responses accompanied by the accumulation of reactive oxygen species (ROS), including hydrogen peroxide (H₂O₂), superoxide (O₂·^−^), singlet oxygen (¹O₂), and hydroxyl radicals (OH·) (Dubey et al., 2014; Ke et al., 2025; Shahid et al., 2014). This causes oxidative damage to major biomolecules (*e.g*., nucleic acids, proteins, sugars, and lipids), disrupts carbon and nitrogen metabolism, and can even trigger cell death, thereby inhibiting plant growth and development (Schlüter et al., 2013; Erdal, 2019; Yang et al., 2019; Liu et al., 2021; Popa, Petrus & Bratu, 2022). To alleviate Cd-induced oxidative damage, plants chelate heavy-metal ions *via* functional groups on the cell wall, such as the hydroxyl and carboxyl groups of hemicellulose/cellulose and the pectic acid groups of pectin (Tang et al., 2023). In parallel, plants bind Cd with free amino acids and other small molecules to form chelates or precipitates (Yang & Huang, 1994). Moreover, when defending against various abiotic stresses, plants can also regulate the proportion of fatty acids to remodel the membrane lipids in the body (Liu et al., 2019). Additionally, plants also cope with Cd stress by synthesizing organic acids that chelate heavy metals and reduce their toxicity (Yang et al., 2005; Dresler et al., 2014).

Metabolomics is used to analyze changes in small-molecule metabolites in samples and to explore differences in metabolic profiles. Among the available detection methods, gas chromatography-time-of-flight mass spectrometry (GC-TOFMS) stands out for its rapid full-spectrum acquisition, high resolution, and wide mass range, offering reliable metabolite identification and enabling the analysis of a broad suite of derivatized metabolites (Cajka & Hajslova, 2004). As a high-performance detection method, GC-TOFMS has become a powerful tool for chemical analysts and has been applied in various fields such as chemistry, geology, biochemistry, pharmacology, medicine, petrochemicals, energy, environmental protection, and food processing (Sheng, Su & Guo, 2006; Medeiros & Simoneit, 2007).

*Salvia miltiorrhiza* Bge. (SM), commonly known as red root or purple Danshen, is a member of the Lamiaceae family and is traditionally used to promote blood circulation and relieve pain associated with blood stasis. Its roots and rhizomes are used to treat liver and spleen enlargement, chest and abdominal pain, ulcer swelling and pain, heart-wrenching pain (Chinese Pharmacopoeia Commission, 2005). Research on SM has overwhelmingly centered on the main medicinal part roots, leaving the non-medicinal organs (especially flowers) largely unexplored. As large-scale cultivation of SM expands, SM flowers are now attracting research interest for their chemical profile and therapeutic potential, including antithrombotic, microcirculatory, hemorheological and neuroprotective effects against cerebral ischemia (Liang & Xu, 2012; Jia et al., 2021; Wang, 2022b). However, the mechanism of Cd resistance in SM flowers has not received sufficient attention. Our previous study with non-targeted metabolomics revealed that SM roots could respond to Cd stress by regulating amino acid metabolism and fatty acid metabolism (Yuan et al., 2022). In this study, in order to reveal the metabolic mechanism underlying the response of SM flowers to Cd stress, SM flowers were taken as the research object, and the metabolic profiles of SM flowers under different levels of Cd stress were analysed using GC-TOFMS. These findings would offer a theoretical basis for the safe cultivation and consumption of SM.

## Materials and Methods

### Experimental design

The SM seeds used in the study were sourced from the SM planting base in Shandong Province. They were identified as SM by Professor Xiaoyun Wang from Jiangxi University of Chinese Medicine. Plump and healthy SM seeds were selected, sowed in pots filled with culture medium (sand: nutrient soil = 1:1), watered every 3 days, and cultivated at room temperature for 6 months. Then, seedlings of a uniform size were selected for the experiment.

Soil (0–20 cm) from the Shennong garden of Jiangxi University of Chinese Medicine was selected. It was naturally air-dried, ground, and passed through a sieve. The pH value and contents of organic matter, total nitrogen (N), total phosphorus (P), total potassium (K), Cd, ammonium nitrogen, nitrate nitrogen, available P, and available K in the soil were measured (Yuan et al., 2022; Fu et al., 2023). The basic physical and chemical properties of the soil in Shennong Garden were as follows: the pH value was 4.6; the organic matter content was 1.97 g·kg^−1^; the contents of total N, total P, and total K were 0.30 g·kg^−1^, 0.28 g·kg^−1^, and 27.15 g·kg^−1^, respectively; the contents of effective N, effective P, and available K were 0.01 g·kg^−1^, 0.01 g·kg^−1^, and 0.08 g·kg^−1^, respectively; the Cd content was 0.92 mg·kg^−1^ (lower than the critical value of Cd (1.0 mg·kg^−1^) that ensured normal plant growth in agricultural and forestry production) (Table S1) (Yuan et al., 2022, 2024).

An aqueous solution of CdCl_2_·2.5H_2_O was prepared and mixed repeatedly with sieved soil to prepare the soil matrix with Cd contents of 0, 25, 50, and 100 mg·kg^−1^, respectively. These groups were named HCK, HT1, HT2, and HT3, respectively. The treatment levels were set based on the study of Zhang et al. (2013), Wei et al. (2020) and Gong et al. (2020). Then, the soil matrix was placed in a pot (with a diameter of 16 cm and a height of 17 cm). After 30 days of soil incubation, the seedlings were transplanted into the pots with one seedling per pot. Each group had three replicates with five seedlings per replicate. The replicates belonged to the repeated technical measurements. After 15 days of growth, samples were collected for metabolomics analysis (Yuan et al., 2022; Fu et al., 2023).

### Metabolome analysis

The metabolome analysis method used in the study was based on our previous research (Yuan et al., 2022; Fu et al., 2023). A series of 50-mg samples were weighed into 2-mL EP tubes and 500 μL of precooled extraction solution (methanol:water volume ratio = 3:1, containing ribosyl alcohol) was added. The samples were then vortexed for 30 s, ground at 40 Hz for 4 min, and sonicated in an ice water bath for 5 min (repeated three times). Subsequently, the samples were placed at 4 °C and centrifuged at 12,000 rpm for 15 min. Then, 200 μL of supernatant was transferred into a 1.5 mL EP tube, and 80 μL of each sample was mixed to form QC samples. The samples were then dried in a vacuum concentrator. Next, 50 μL of methoxyamine salt reagent (methoxyamine hydrochloride, dissolved in 20 mg/mL pyridine) was added to the dried metabolites, gently mixed, and incubated in an 80 °C oven for 30 min. Then, 70 μL of N,O-Bis (trimethylsilyl) trifluoroacetamide (BSTFA), which contained 1% trimethylchlorosilane (TMCS, v/v), was added to each sample, and the mixture was incubated at 70 °C for 1.5 h. Finally, the mixture was cooled to room temperature, and 5 μL of fatty acid methyl esters (FAMEs, dissolved in chloroform) was added for detection.

Sample detection was performed using an Agilent 7890 GC-TOFMS system, with an Agilent DB-5MS capillary column (30 m × 250 μm × 0.25 μm, J&W Scientific, Folsom, CA, USA). 1 μL of each sample was injected in non-split mode; helium was used as the carrier gas, the purge flow rate was 3 mL·min^−1^ through the spacer, and the gas flow rate was 1 mL·min^−1^ through the column. The initial temperature was maintained at 50 °C for 1 min, raised to 310 °C at a rate of 10 °C·min^−1^, and held at 310 °C for 8 min. The temperatures of the injection port, transmission line, and ion source were 280 °C, 280 °C, and 250 °C, respectively, and the ionization voltage was −70 eV. After a solvent delay of 6.4 min, mass spectrometry data were collected in full scan mode at a rate of 12.5 spectra per second in the m·z^−1^ range of 50–500.

### Data analysis

The mass spectrometry data were analysed with ChromaTOF software (V 4.3x, LECO) for peak extraction, baseline correction, deconvolution, peak integration, and peak alignment (Kind et al., 2009). The LECO Fiehn Rtx5 database was used for the qualitative characterization of substances, including mass spectrometry matching and retention time index matching. Finally, peaks with detection rates below 50% or relative standard deviation (RSD) > 30% from the QC samples were removed (Dunn et al., 2011).

In order to reduce the impact of detection system errors on the results, series of raw data were prepared and organised. The main steps included: (1) Deviation filtering: filtering individual peaks to remove noise, and filtering deviation values based on RSD and coefficient of variation (CV); (2) Missing value filtering: filtering individual peaks and only retaining peak area data with a single group having less than 50% null values or all groups having less than 50% null values; (3) Missing value imputation: simulation of missing values in the original data with a numerical simulation method of filling in at least half of the minimum value; (4) Data normalization: normalization using internal standards (IS). Finally, 371 peaks were retained.

Firstly, clustering analysis was performed on the pre-processed data, and a clustering heatmap was drawn. All metabolites were classified, and a pie chart was drawn. The differences in metabolites between the control group and each Cd-stressed group were analysed by one-way ANOVA. Secondly, unsupervised principal component analysis (PCA) and supervised orthogonal partial least squares discriminant analysis (OPLS-DA) were performed to analyse the sample distribution, and the variable importance in the projection (VIP) of the first principal component was obtained using the OPLS-DA model. The validity of the OPLS-DA model was tested using a 200-fold permutation test, with R2 > 0.7 and Q2 > 0.4 in the permutation test, indicating the effectiveness of the OPLS-DA model (Yuan et al., 2022). Identified differential metabolites (DMs) were screened based on the *FDR* (*p* value adjusted using False Discovery Rate) and VIP value (*FDR* < 0.05 and VIP > 1). Finally, the pathway analysis of the DMs was conducted, and differential metabolic pathways were selected based on *FDR* < 0.05 and Impact > 0.1.

The PCA and OPLS-DA models were tested using SIMCA software (V16.0.2, Sartorius Stedim Data Analytics AB, Umea, Sweden). One-way ANOVA was performed using IBM SPSS Statistics 20.0. The pie charts were created using R (ggplot2, 3.3.5). The pathway analysis was conducted with MetaboAnalyst 6.0 (https://www.metaboanalyst.ca). All data were standardized before analysis.

## Results

### Quality control

**Figure 1:**
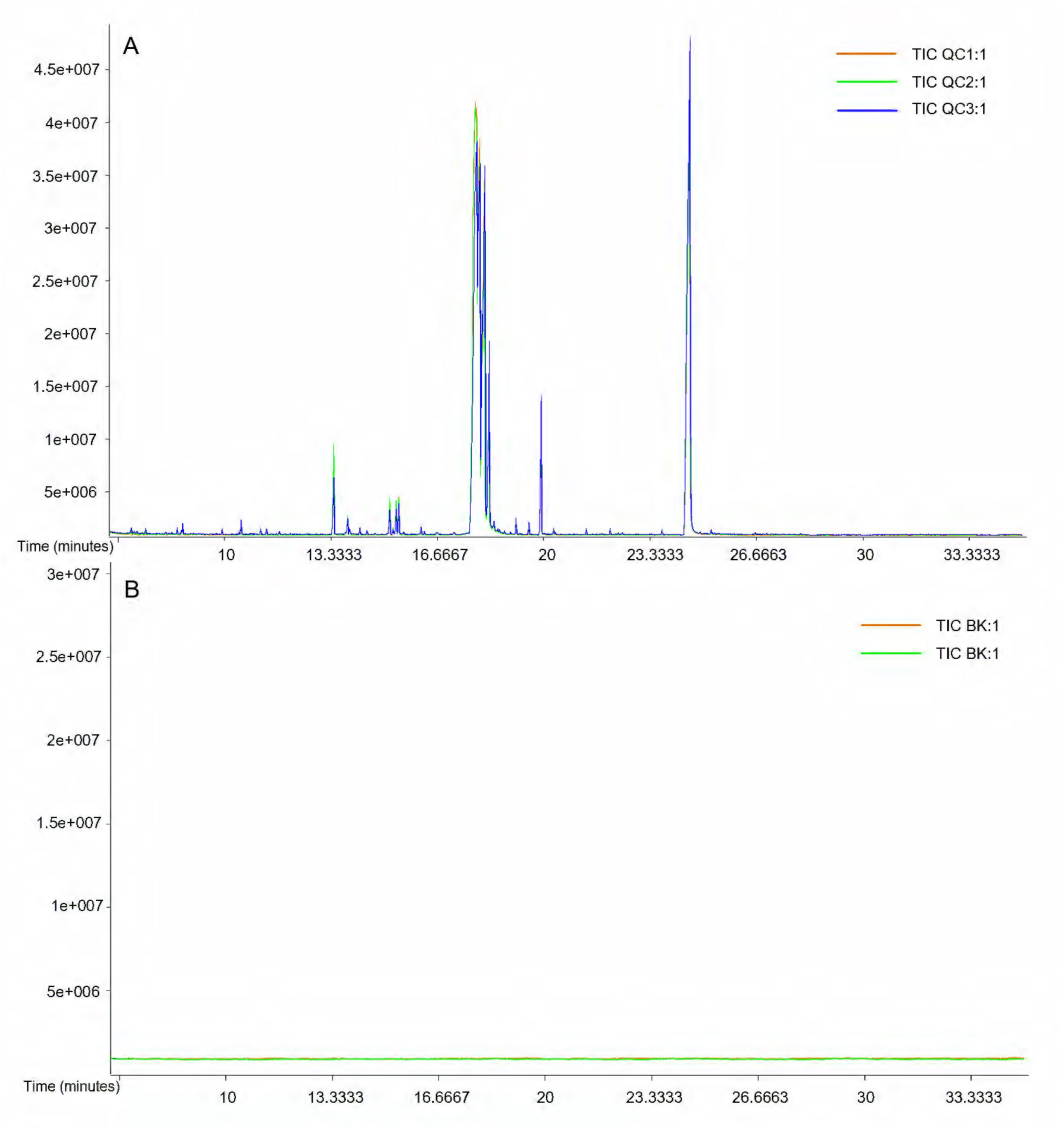
Total ion chromatography (TIC) of samples. (A) TIC of the quality control samples; (B) TIC of the quality control for the blank sample.

The retention time and peak area of the QC sample and the total ion chromatogram (TIC) peak overlapped well, indicating that the instrument was stable (Fig. 1). The RSD of the peak area of the internal standard in the QC sample was 15.86%, which was less than 30%, indicating excellent system stability and reliable data acquisition (Table S2). No significant peaks were detected in the blank sample, confirming the absence of cross-contamination and ensuring data reliability (Fig. 1). All the correlations between the QC samples were above 0.9 (Fig. S1). This indicated that the entire method was stable and the data quality was high.

### Metabolite profiles of SM flowers under different contents of Cd stress

**Figure 2:**
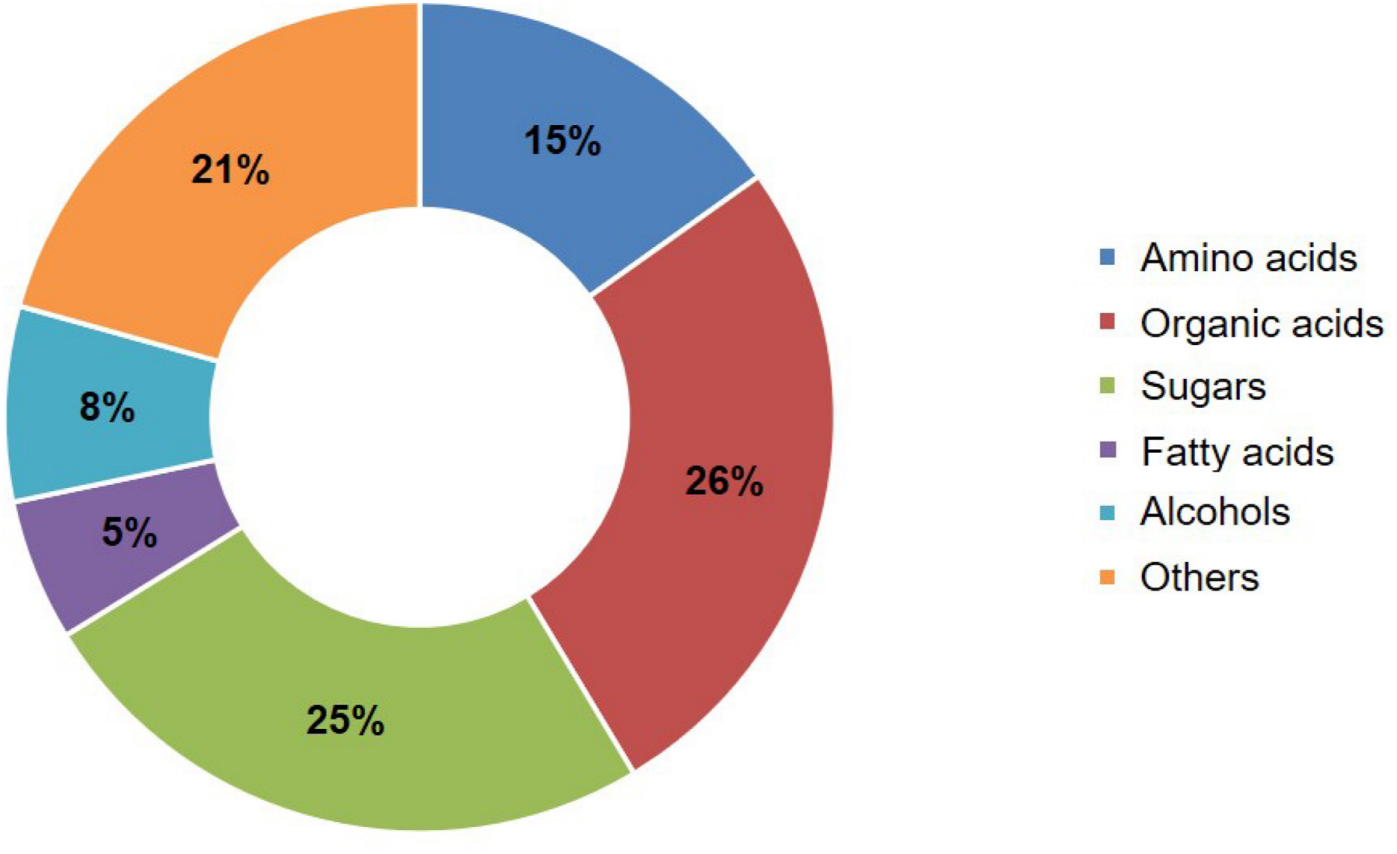
Relative abundances of classified metabolite categories in SM flowers with different levels of Cd stress. The different coloured blocks represented different classification categories, and the percentage represented the percentage of metabolites belonging to that type among all identified metabolites.

The metabolites were divided into six categories: amino acids, organic acids, sugars, lipids, alcohols, and others (Fig. 2; Table S3). Among them, organic acids accounted for the largest proportion at 26.21%, followed by sugars at 24.83%; amino acids accounted for 15.17%, while lipids accounted for 5.52% (Fig. 2).

### Differential metabolites of SM flowers under different contents of Cd stress

**Figure 3:**
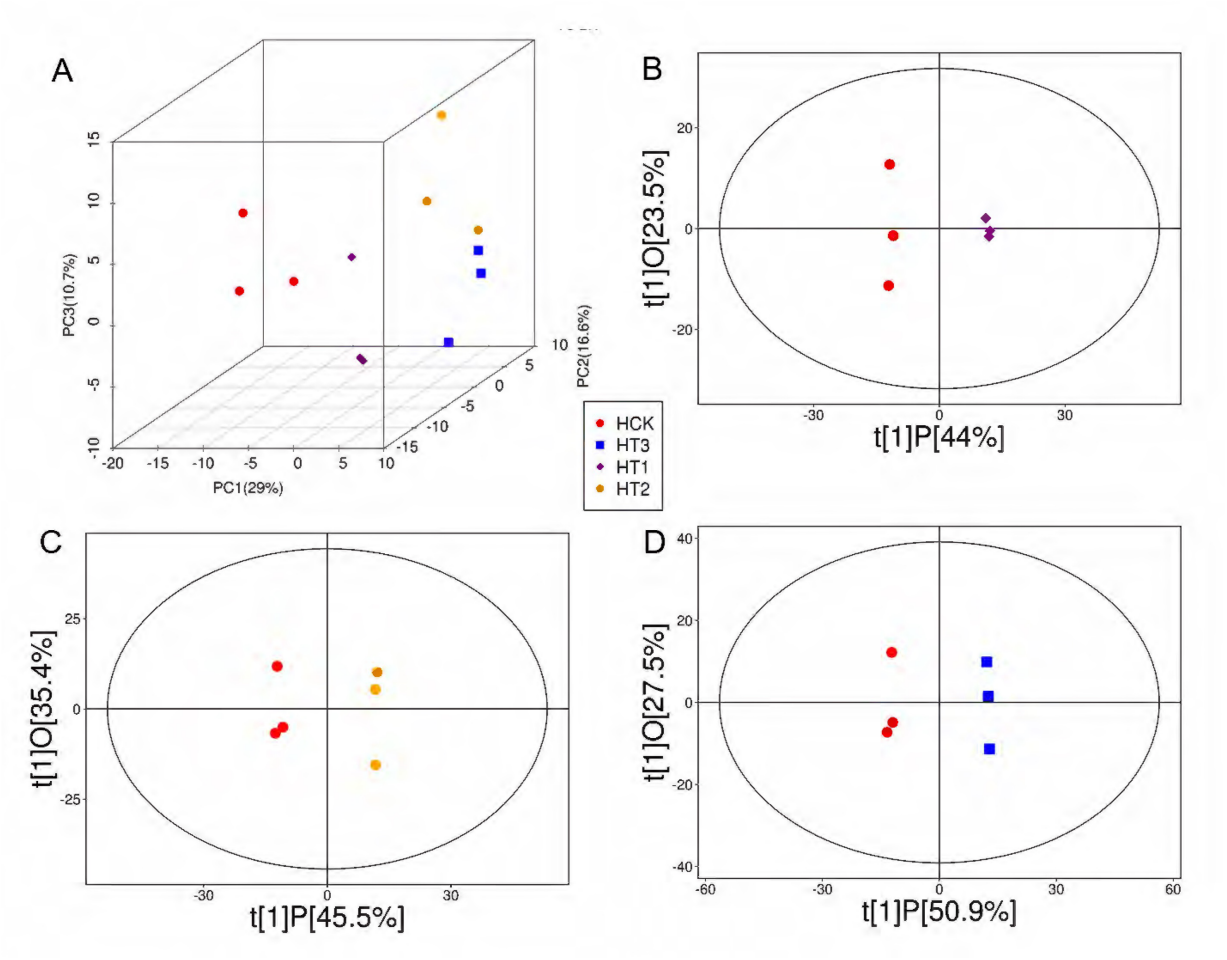
Trajectories of PCA (A) and OPLS-DA (B–D) highlights level-dependent metabolic shifts along gradient in SM flowers. (A) The horizontal axis, vertical axis, and perpendicular axis correspond to PC1, PC2, and PC3, respectively, with variance explained percentages of 29%, 6.6%, and 0.7%, respectively; (B–D) the horizontal axis t[1]P represented the predicted principal component score of the first principal component, showing the differences between sample groups, and the vertical axis t[1]O represented the orthogonal principal component score, showing the differences within sample groups; each scatter represented a sample, and scatters of different colour and shape represented different groups.

According to the PCA results, all samples were located in the 95% confidence interval (Hotelling’s T-Squared ellipse), and the control group and each Cd treatment group were significantly distinguished (Fig. 3A). According to the results of the reliable OPLS-DA model (Fig. S2), the samples of each group were clustered together, and the samples between groups were clearly distinguished (Figs. 3B–3D).

**Figure 4:**
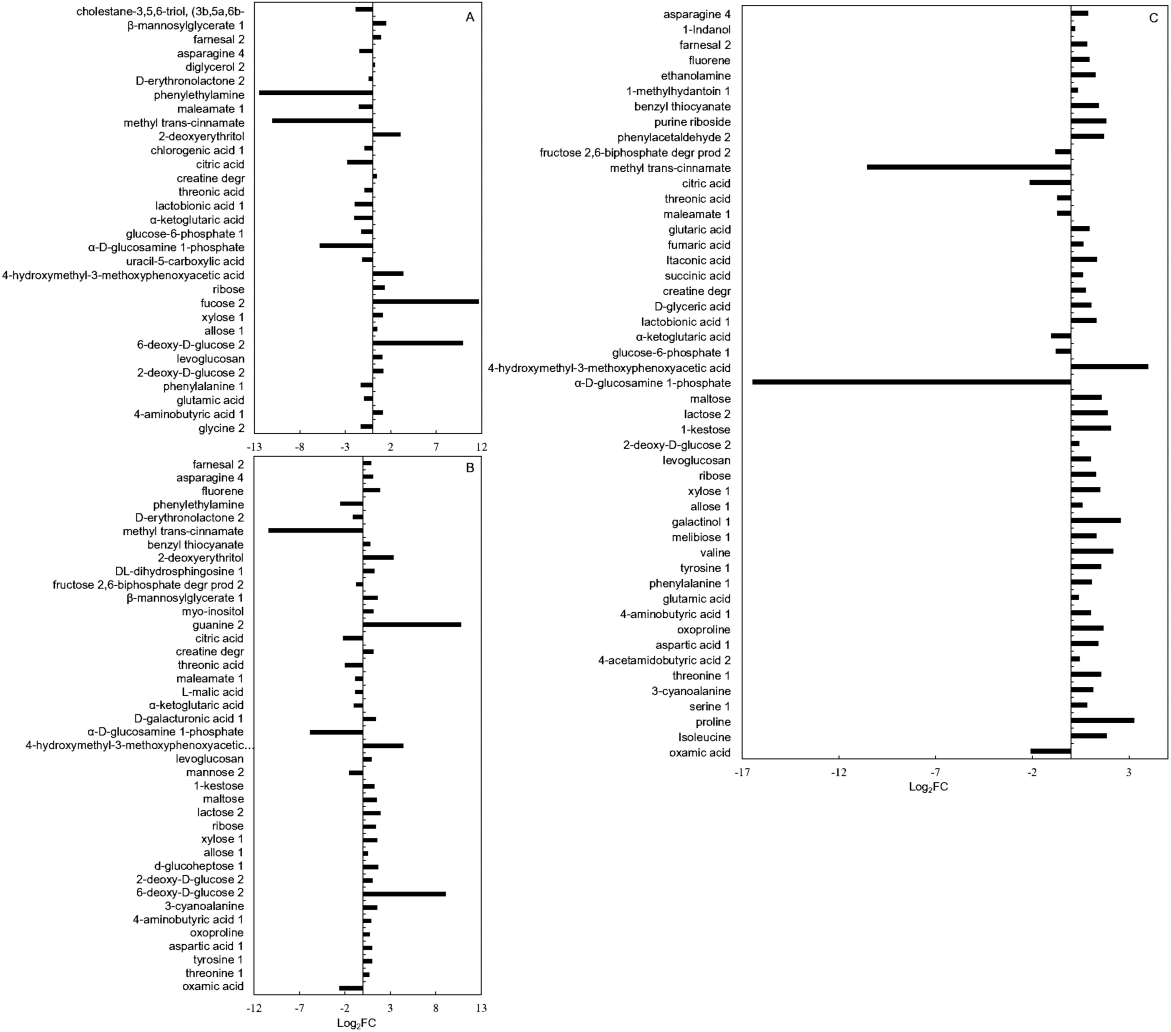
All identified DMs in SM flowers with different levels of Cd stress. (A), (B) and (C) represented the changes of DMs contents in HT1, HT2, and HT3, respectively; FC represented ratios of DMs contents between Cd stressed group and control group.

**Figure 5:**
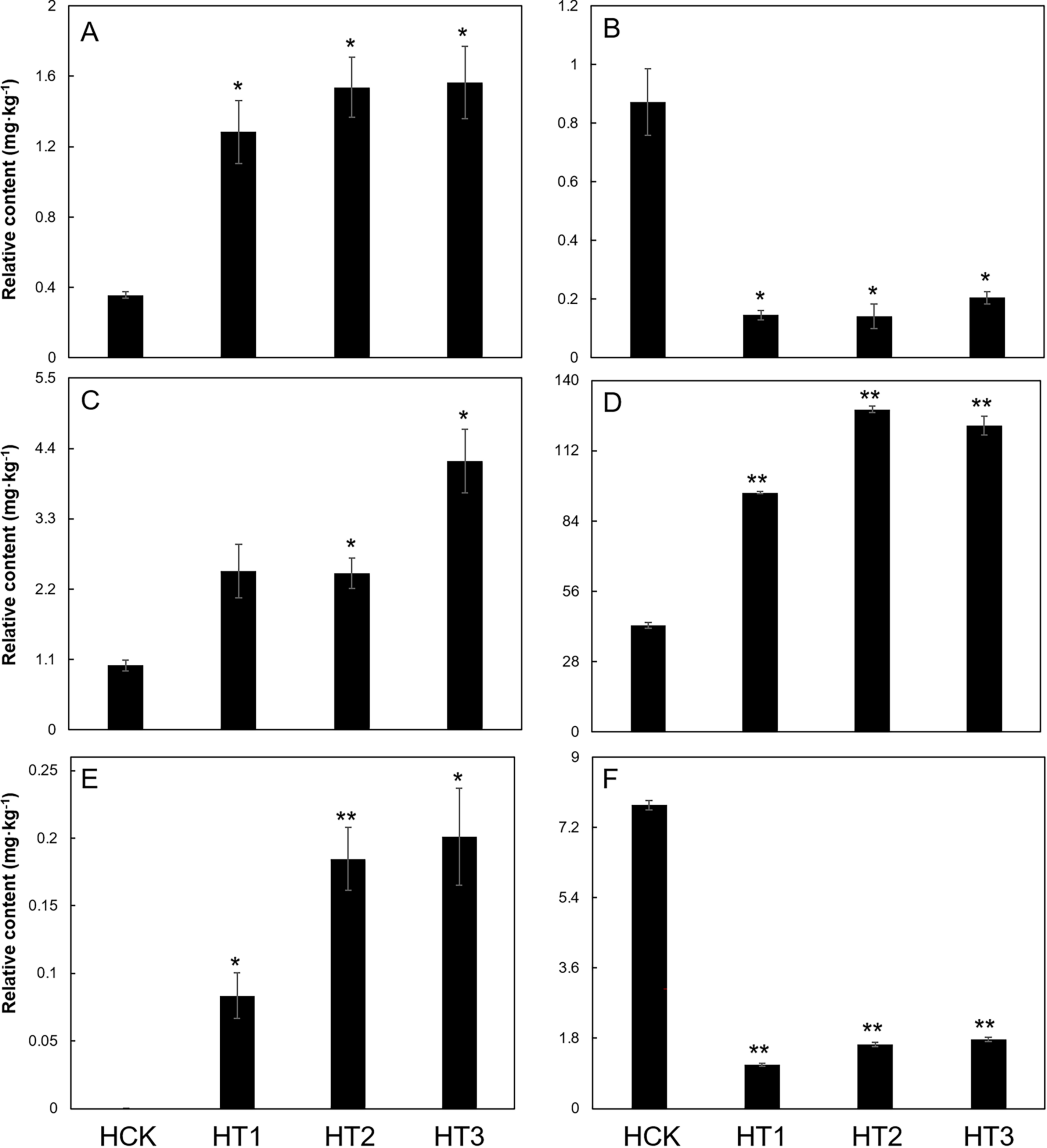
Relative contents of selected metabolites with great changes between the control group and the Cd stressed groups. (A)–(F) represented relative content of tryptophan, oxamic acid, 1-kestose, xylose, arachidic acid, and citric acid in SM flowers with different levels of Cd stress, respectively; the asterisk indicated a significant difference compared to HCK; *p* values were corrected by FDR; *, 0.01 < *FDR* < 0.05; **, 0.001 < *FDR* < 0.01; vertical bars above the columns indicate the standard error of each mean (mean ± SE).

DMs between the control group and each Cd-treated group were screened based on *FDR* < 0.05 and VIP > 1 (Table S4). There were significant differences in the DMs among the different Cd treatment groups. There were 31, 40, and 49 DMs in HT1, HT2, and HT3, respectively (Fig. 4; Table S4). Among all the metabolites, oxamic acid and citric acid contributed most to the down-regulated amino acids and organic acids, respectively; their levels in HCK were over four times those of the Cd stressed groups. Tryptophan and arachidic acid contributed most to the up-regulated amino acids and organic acids, respectively; compared with HCK, tryptophan levels increased by more than twice, and arachidic acid levels improved by more than 200 times in all Cd-stressed groups. Xylose and 1-kestose contributed most to the up-regulated sugars; their levels in all Cd stressed groups were more than twice those of the control (Figs. 4, 5).

### Main pathways enriched by DMs

**Figure 6:**
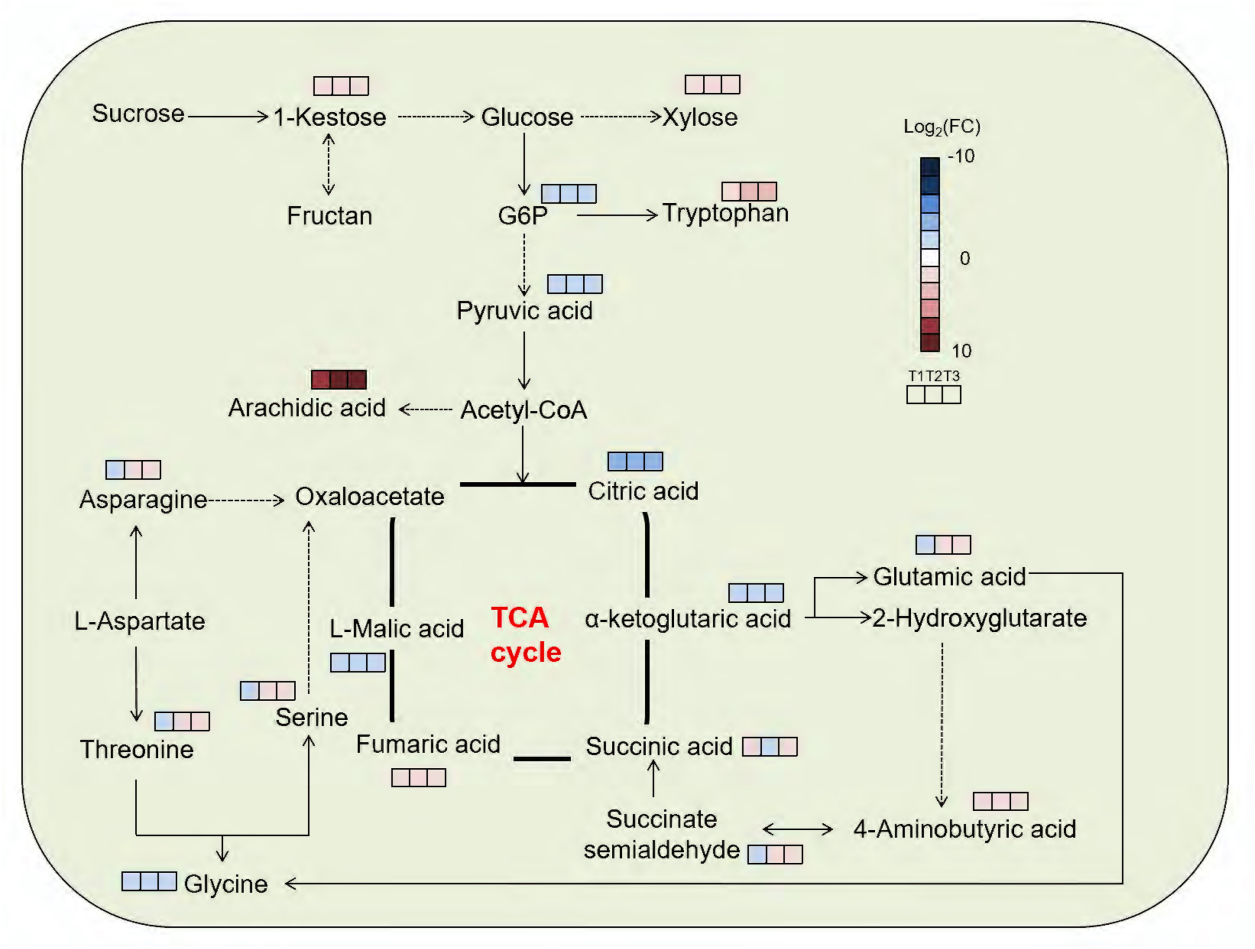
Metabolic map of SM flowers based on GC-TOFMS with different levels of Cd stress. Log2(FC), the log2-transformed fold change in flower metabolite abundance of the Cd stress group relative to the control group; red indicates upward adjustment, blue indicates downward adjustment; the dashed line represents multiple steps, while the straight line represents one step.

According to the enrichment analysis results of differential metabolic pathways (*FDR* < 0.05), DMs in HT1 were significantly enriched in alanine, aspartate, and glutamate metabolism, glyoxylate and dicarboxylic acid metabolism, and butanoate metabolism (Fig. 6; Table 1). In HT2, only alanine, aspartate and glutamate metabolism was enriched, whereas HT3 showed significant enrichment in glyoxylate and dicarboxylic acid metabolism, butanoate metabolism, TCA cycle, and glycine, serine and threonine metabolism (Fig. 6; Table 1).

**Table 1:** Results of pathway analysis involving all the differential metabolites in SM flowers with different levels of Cd stress.

Group	Pathway	Total Cmpd	Expected	Hits	FDR	Impact
**T1**	Alanine, aspartate and glutamate metabolism	22	0.2030	4	0.0028	0.5252
	Glyoxylate and dicarboxylate metabolism	29	0.2676	4	0.0044	0.2181
	Butanoate metabolism	17	0.1569	3	0.0121	0.1364
**T2**	Alanine, aspartate and glutamate metabolism	22	0.2755	4	0.0103	0.3273
**T3**	Alanine, aspartate and glutamate metabolism	22	0.4061	7	0.0000	0.6547
	Glyoxylate and dicarboxylate metabolism	29	0.5353	6	0.0004	0.1883
	Butanoate metabolism	17	0.3138	4	0.0055	0.1364
	Citrate cycle (TCA cycle)	20	0.3692	4	0.0068	0.2460
	Glycine, serine and threonine metabolism	33	0.6091	4	0.0303	0.3242

**Note:**

All pathways with *FDR* < 0.05 and Impact > 0.1 selected for KEGG enrichment analysis were shown in the table; Total Cmpd, total number of compounds in the pathway; Hits, the number of actually matched compound in the pathway; FDR, *p* value adjusted using False Discovery Rate; Impact, pathway impact value.

## Discussion

Amino acids, sugars, and organic acids play important roles in the response of SM roots to Cd stress (Yuan et al., 2022). In this study, amino acids, sugars, and organic acids also played crucial roles in the response of SM flowers to Cd stress (Fig. 6; Table 1).

### Amino acids play major roles in the response to different levels of Cd stress

Amino acids function as osmotic regulators in cell membranes; on the one hand, they mediate plant responses to Cd stress, and on the other hand, they serve as key precursors of protective compounds (*e.g*., second metabolites) that enhance Cd tolerance (Zhu et al., 2022; Fu et al., 2023) (Fig. 6; Table 1). In the study, oxamic acid and tryptophan were identified as vital contributors to Cd resistance in SM flowers. Firstly, oxamic acid contributed most among the down-regulated differential amino acids across Cd treatments (*FDR* < 0.05) (Fig. 5). Oxamic acid belonged to the free amino acids, which could act as osmotic regulators in cell membranes during plant responses to Cd stres**s** (Liang et al., 2021). Functional groups such as carboxyl and amino groups of free amino acids could form coordination bonds with Cd ions, thereby reducing the concentration of free Cd ions and mitigating their toxicity to organisms (Yu et al., 2022). This might lead to the downregulation of free amino acid (*e.g*., oxamic acid) (Figs. 4, 5). Unlike our results, oxamic acid content increased significantly under low-temperature stress (Liu, 2024). This opposing trend underscores the context-dependent roles of free amino acids: whereas cold stress prompts plants to accumulate osmotic protectants such as oxamic acid to stabilize cell membranes, Cd stress in our study led to its marked decline (Liu, 2024) (Figs. 4, 5).

Secondly, tryptophan contributed most among the up-regulated differential amino acids in all groups (HT1, HT2, and HT3) (*FDR* < 0.05) (Fig. 5). Tryptophan, an aromatic amino acids, was an important precursor of secondary metabolites (*e.g*., alkaloids, indoleacetic acid, phytoalexin) that play important roles in Cd resistance (Stepanova et al., 2008; Tao et al., 2008; Wang et al., 2015). Tryptophan application could effectively alleviate Cd toxicity in plants (Yu et al., 2018; Godoy et al., 2021; Kou, Tran & Dai, 2024). For example, elevated intracellular tryptophan could enhance Cd tolerance in *Arabidopsis thaliana* (Sanjaya et al., 2008), and tryptophan treatment could mitigate Cd stress in *A. thaliana* (Li & Qi, 2023) and *Brassica oleracea* (Jiang, 2021). The observed up-regulation of tryptophan in the present study might serve to produce some secondary metabolites to counteract Cd exposure. Interestingly, the change trend in the tryptophan content of SM flowers in the study differed from that of SM roots. In SM roots, the tryptophan content increased at first and then decreased with the increase of the soil Cd content (Yuan et al., 2022). This was related to differential distribution of metabolites in different tissues of SM, which was stemmed from tissue-specific gene expression in SM (Li et al., 2020; Lu et al., 2024; Zhang et al., 2021a).

### Sugars play major roles in the response to different levels of Cd stress

Sugars such as xylose and 1-kestose contributed significantly to Cd resistance in SM flowers. In the present study, xylose levels in Cd stressed groups were more than twofold higher than in HCK (Figs. 4, 5). Xylose was a widespread plant sugar that occured mainly as xylan (Wang et al., 2018; Gao et al., 2020). Xylan was a major constituent of hemicellulose, which served as the primary site for heavy-metal sequestration in the plant cell wall (Chen et al., 2013; Li et al., 2019b). Consistent with the study, the xylose content in hemicellulose of SM roots increased under Cd stress (Liao et al., 2025). The up-regulation of xylose in Cd-stressed groups suggested that Cd stress affected the structure of cell wall in SM flowers. This was related to that plants could increase the metal ion binding capacity of the cell wall by enhancing hemicellulose content, thereby reducing the entry of metal ions into the cytoplasm (Wang, 2022a).

1-kestose was a key intermediate in the irreversible conversion of sucrose to fructan (Valluru & den Ende, 2008) (Fig. 6). In this study, the 1-kestose content in all Cd stressed groups were above twofold higher than in HCK, neither fructan nor sucrose was identified among the DMs (Figs. 4, 5). This illustrated that Cd hold an impact on the sugar metabolism in SM flowers. Similarly, 1-kestose content in *Poa pratensis* significantly increased under low temperature (Zhao et al., 2020). Fructan could accumulate in plants during moderate stress, but as stress intensified, it was degraded to sustain plant growth (Wang, 2000). Meanwhile, under adverse conditions, fructan could be degraded into monosaccharides with the increase of sugar concentration in vacuole sap, and this lowered the osmotic potential and enhanced plant-cell stress tolerance (Turner & Kramer, 1980; Valluru & den Ende, 2008). All these might result in the upregulation of 1-kestose in Cd stressed groups (Fig. 5).

### Organic acids play major roles in the response to different levels of Cd stress

Organic acids (especially arachidic acid and citric acid) acted important roles in Cd resistance in SM flowers. Arachidic acid was a kind of fatty acids. In the study, arachidic acid contributed most to the down-regulation of organic acids (Fig. 5). Several studies have shown that the qualitative and quantitative composition of fatty acids in plants changed under heavy metal stress (Jemal, Zarrouk & Ghorbal, 2000; Kai et al., 2012; Liu et al., 2019). Plants would respond to heavy metal stress by altering cell-membrane fluidity, a process mediated by the unsaturated fatty acid (UFA) content. During the process, fatty acid desaturases (FADs) catalysed the formation of double bonds at specific positions of saturated fatty acid (SFA) chains to produce UFAs (Li et al., 2019a). While SFA arachidic acid (C20:0) content increased, and the UFA linolenic acid (C18:3) decreased under Cd stress in SM flowers (Fig. 5). Inconsistent with the phenomenon, under Cd stress, the content of UFA (*e.g*., C18:1 and C18:2) increased, and the content of SFA (*e.g*., C18:0) decreased in SM roots (Yuan et al., 2022). This suggested that Cd stress affected the cell membrane structures of SM flowers. This also indicated that there were inter-organizational differences in the mechanisms of coping with Cd stress (Liang, Wang & Chen, 2024). The differences in Cd-stress responses between SM roots and flowers might result from tissue-specific regulation of response-gene expression (He et al., 2022; Li et al., 2020; Lu et al., 2024, Zhang et al., 2021a).

Citric acid was an important intermediate product of the TCA cycle. In the study, citric acid contributed most to the down-regulation of organic acids (Figs. 4, 5). Some studies have reported that citric acid significantly increased the absorption of heavy metals (*e.g*., Cd, Cu, Zn, Pb) by plants and promoted their transport and accumulation (Ni, 2004; Han, 2005). Similarly, the citric acid content in *Poa pratensis* leaves decreased under Cd stress (Wang et al., 2026). While the citric acid content of *Abutilon theophrasti Medicus* increased under Cd stress (Yang, 2024). This might be because different plants could synthesize different organic acids to cope with heavy metal stress (Yang, 2011). In contrast to the findings in SM flowers, citric acid in SM roots was down-regulated under both mild and moderate Cd stress but up-regulated under severe Cd stress (Yuan et al., 2022). These findings indicated that different plant tissues synthesized different organic acids to cope with Cd stress (Yang, 2024). These also reflected tissue-specific regulation of Cd response genes in SM roots and flowers under Cd stress (He et al., 2022; Li et al., 2020; Lu et al., 2024, Zhang et al., 2021a).

## Conclusion

Medicinal plant resources can be contaminated by heavy metals, posing a threat to human beings. In this study, SM flowers were sampled, and their metabolites were measured with GC-TOFMS.

The results of PCA and the reliable OPLS-DA model revealed that the samples of each group were clustered together, and the samples between the groups were clearly distinguished. This indicates that Cd stress significantly affected the metabolite profiles of SM flowers. Second, the number of DMs differed between the Cd-treated groups, and the DM pathways differed among the HT1, HT2, and HT3 groups. This suggests that SM flowers adopt distinct metabolic adjustments in response to varying Cd stress levels. Third, DMs were mainly enriched in amino acid metabolism (including alanine, aspartate, and glutamate metabolism, glycine, serine and threonine metabolism), carbon metabolism (TCA cycle), and secondary metabolism (including glyoxylate and dicarboxylic acid metabolism, butanoate metabolism). This indicates that SM flowers primarily regulate carbon, nitrogen, and secondary metabolism to resist Cd stress.

Finally, oxamic acid and citric acid contributed most among the down-regulated amino acids and organic acids, respectively; tryptophan and arachidic acid contributed most among the up-regulated amino acids and organic acids, respectively; xylose and 1-kestose contributed most among the up-regulated sugars. These suggest that leveraging these metabolites, SM flowers resist Cd by remodeling cell-wall architecture, adjusting membrane fluidity, and fine-tuning intracellular free substances. Besides, these findings indicate that Cd stress reshapes the metabolism of SM flowers in a pattern distinct from that reported for roots. All these findings enable the safe cultivation of SM and the breeding of low-Cd-accumulating varieties by targeting elevated levels of specific metabolites (*e.g*., citric acid, tryptophan). These also highlight the need for organ-targeted strategies to limit Cd translocation and ensure medicinal product safety. Nevertheless, the study was constrained by its untargeted metabolomics approach and single-time-point sampling, and the metabolic mechanism of SM flowers in response to Cd stress should be further investigated.

## Supplemental Information

10.7717/peerj.21149/supp-1 Supplemental Information 1 Correlation analysis of QC samples.

10.7717/peerj.21149/supp-2 Supplemental Information 2 Permutation plot test of OPLS-DA models.

10.7717/peerj.21149/supp-3 Supplemental Information 3 Basic physical and chemical indexes of the pristine soil. Raw data for soil analyses

10.7717/peerj.21149/supp-4 Supplemental Information 4 Response of internal standards.

10.7717/peerj.21149/supp-5 Supplemental Information 5 All metabolites detected in SM flowers with different levels of Cd stress.

10.7717/peerj.21149/supp-6 Supplemental Information 6 Differential metabolites in SM flowers with different levels of Cd stress.

## Additional Information and Declarations

### Data Availability

The following information was supplied regarding data availability:

The raw data for soil analyses is available in the Supplemental File.

The metabolomics raw data is available at OMIX, China National Center for Bioinformation: OMIX011376.
