# Supplementary figures and images for: Gas chromatography-time-of-flight mass spectrometry (GC-TOFMS)-based metabonomic response of Salvia miltiorrhiza flowers to cadmium stress
Source: PeerJ. 2026 Apr 27;14:e21149. doi: 10.7717/peerj.21149 (PMC13131354; doi:10.7717/peerj.21149)

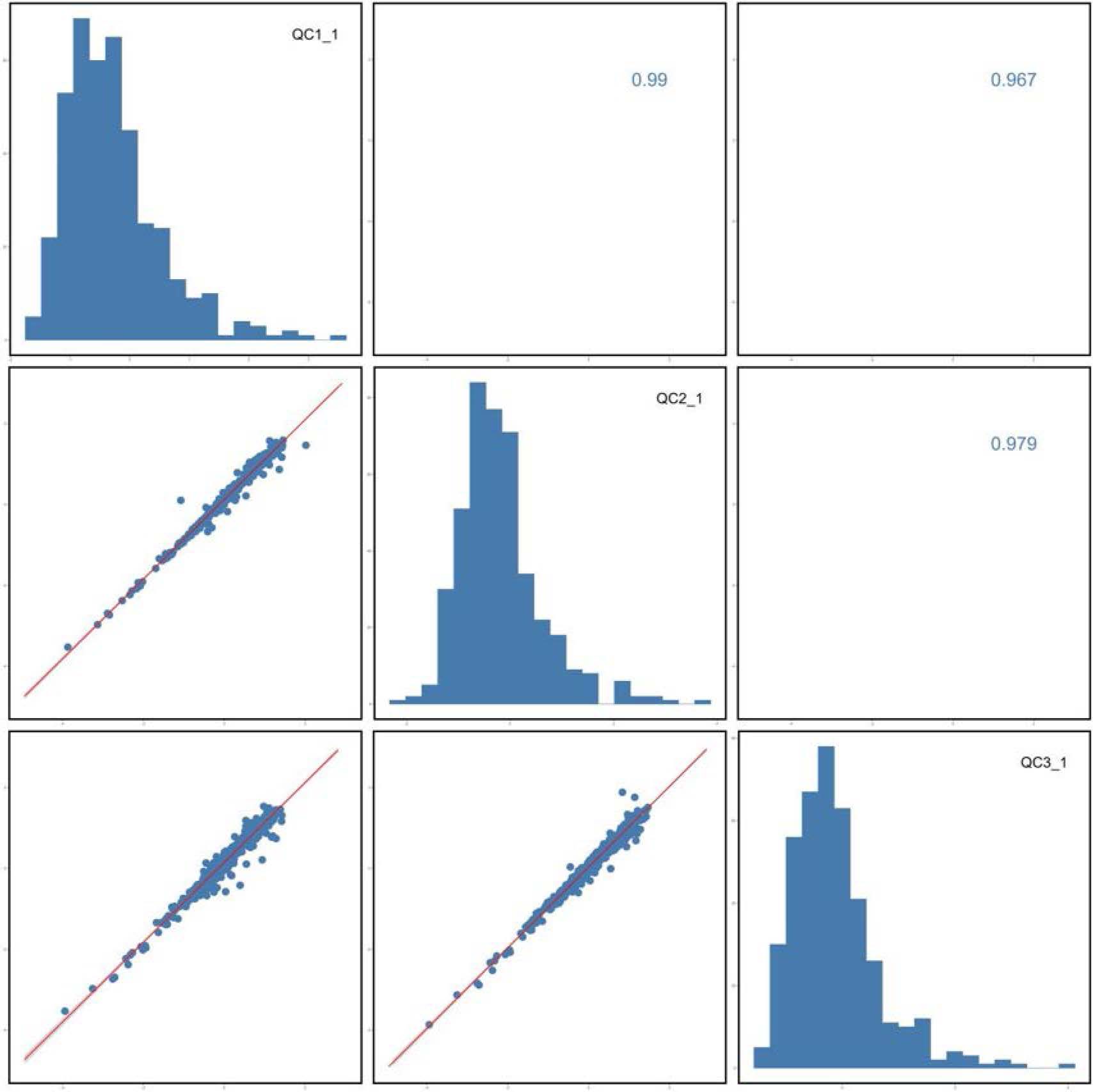

Supplement: Supplemental Information 1 [file peerj-14-21149-s001.jpg]

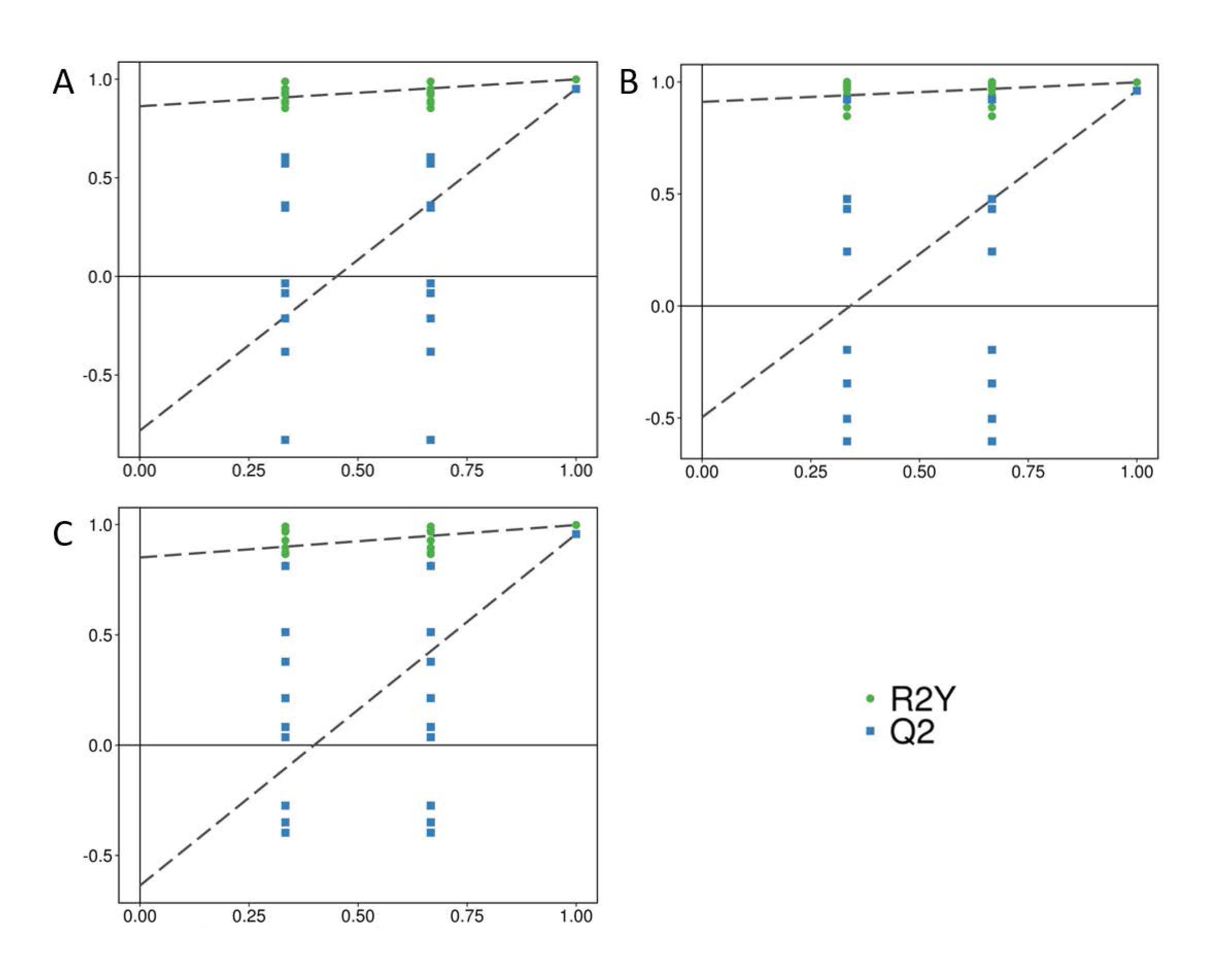

Supplement: Supplemental Information 2 [file peerj-14-21149-s002.jpg]
