# Supplementary material for: Gas chromatography-time-of-flight mass spectrometry (GC-TOFMS)-based metabonomic response of Salvia miltiorrhiza flowers to cadmium stress
Source: PeerJ. 2026 Apr 27;14:e21149. doi: 10.7717/peerj.21149 (PMC13131354; doi:10.7717/peerj.21149)
Supplement: Supplemental Information 4 [file peerj-14-21149-s004.docx]

Table S2 Response of internal standards.

| Sample | Area |
| --- | --- |
| QC1 | 141489.797 |
| QC2 | 110151.488 |
| QC3 | 107286.565 |
| Average | 119642.6167 |
| RSD | 15.86% |

RSD, relative standard deviation; QC, quality control sample.
