# Supplementary material for: Gas chromatography-time-of-flight mass spectrometry (GC-TOFMS)-based metabonomic response of Salvia miltiorrhiza flowers to cadmium stress
Source: PeerJ. 2026 Apr 27;14:e21149. doi: 10.7717/peerj.21149 (PMC13131354; doi:10.7717/peerj.21149)
Supplement: Supplemental Information 5 [file peerj-14-21149-s005.docx]

Table S3 All metabolites detected in SM flowers under different content of Cd stress.

| No. | Metabolite | RT (s) | Mass | HCK (mg kg^-1^) | HT1 (mg kg^-1^) | HT2 (mg kg^-1^) | HT3 (mg kg^-1^) |
| --- | --- | --- | --- | --- | --- | --- | --- |
| 1 | Analyte34 | 6.4321 | 73 | 5.3757±1.4723 | 6.3255±0.6408 | 9.1594±1.8823 | 9.1903±1.8893 |
| 2 | Analyte 5 | 6.4512 | 129 | 1.2483±0.1949 | 0.9984±0.1350 | 1.5563±0.2426 | 1.4109±0.2597 |
| 3 | Analyte 6 | 6.4691 | 221 | 0.2773±0.0443 | 0.1199±0.0650 | 0.3013±0.0423 | 0.3445±0.0589 |
| 4 | Analyte 7 | 6.4812 | 87 | 0.1140±0.1180 | 0.3456±0.0413 | 0.4689±0.0734 | 0.5129±0.0957 |
| 5 | Analyte 9 | 6.5307 | 95 | 1.2653±0.2956 | 1.1165±0.0802 | 1.3887±0.2373 | 1.3948±0.2744 |
| 6 | Analyte 11 | 6.5555 | 98 | 0.6452±0.1658 | 0.5601±0.0195 | 0.7779±0.1473 | 0.7079±0.1170 |
| 7 | Analyte 12 | 6.5683 | 82 | 0.4393±0.1110 | 0.0405±0.0419 | 0.1033±0.1069 | 0.1654±0.0932 |
| 8 | Analyte 13 | 6.5995 | 160 | 0.6573±0.1491 | 0.9206±0.0527 | 1.1188±0.2145 | 1.2041±0.1985 |
| 9 | Analyte 15 | 6.6427 | 147 | 0.2421±0.0505 | 0.2579±0.0114 | 0.2849±0.0529 | 0.2731±0.0275 |
| 10 | Analyte 16 | 6.6622 | 54 | 0.0002±0.0000 | 0.0001±0.0000 | 0.2140±0.1119 | 0.3844±0.0726 |
| 11 | Analyte 17 | 6.6733 | 69 | 0.1160±0.1201 | 0.0860±0.0890 | 0.3136±0.1017 | 0.2520±0.0342 |
| 12 | Analyte 18 | 6.6927 | 117 | 0.1914±0.0367 | 0.1529±0.0142 | 0.1789±0.0349 | 0.1735±0.0408 |
| 13 | Analyte 20 | 6.7364 | 121 | 0.0725±0.0427 | 0.1286±0.0166 | 0.0866±0.0544 | 0.0472±0.0487 |
| 14 | Analyte 21 | 6.7662 | 281 | 1.4848±0.3032 | 1.5252±0.0721 | 1.8887±0.3736 | 1.8689±0.2896 |
| 15 | Analyte 24 | 6.8642 | 248 | 0.6042±0.1391 | 0.6432±0.0439 | 0.8407±0.1662 | 0.8118±0.1219 |
| 16 | Analyte 25 | 6.8550 | 71 | 0.6369±0.3468 | 0.0704±0.0729 | 0.6580±0.0871 | 0.4562±0.0779 |
| 17 | Analyte 26 | 6.9213 | 71 | 0.7526±0.1599 | 0.4466±0.0514 | 0.5902±0.1147 | 0.5625±0.0959 |
| 18 | Analyte 27 | 6.9404 | 73 | 3.4013±0.8916 | 5.0262±0.2859 | 5.2650±1.1910 | 5.4349±0.8862 |
| 19 | Analyte 28 | 6.9517 | 89 | 0.1531±0.0343 | 0.2133±0.0114 | 0.2346±0.0393 | 0.1346±0.0732 |
| 20 | Analyte 29 | 6.9694 | 93 | 0.8046±0.2679 | 0.6805±0.0231 | 1.0126±0.2151 | 0.9324±0.1725 |
| 21 | Analyte 31 | 7.0175 | 57 | 1.3848±0.3059 | 1.3253±0.1126 | 1.7079±0.3072 | 1.7375±0.2729 |
| 22 | Analyte 32 | 7.0212 | 114 | 0.6997±0.1378 | 0.7139±0.0611 | 0.9360±0.1755 | 0.9039±0.1148 |
| 23 | Analyte 33 | 7.0490 | 248 | 2.8176±0.6721 | 2.9824±0.2184 | 3.8220±0.8164 | 3.7737±0.5760 |
| 24 | Analyte 34 | 7.0724 | 147 | 16.7867±3.9530 | 17.0202±1.4184 | 22.9124±5.0171 | 19.7428±3.1238 |
| 25 | Analyte 36 | 7.1276 | 141 | 0.5921±0.1440 | 0.5293±0.0558 | 0.6718±0.1527 | 0.5695±0.0831 |
| 26 | Analyte 37 | 7.1596 | 207 | 7.9579±1.9257 | 8.2194±0.3870 | 10.6198±2.4139 | 9.7103±1.5235 |
| 27 | 2-hydroxypyridine | 7.2453 | 152 | 15.4977±4.2492 | 18.5011±1.3491 | 22.0265±5.1467 | 21.9719±2.9543 |
| 28 | Analyte 40 | 7.2484 | 318 | 0.0002±0.0000 | 1.7479±0.9205 | 0.0002±0.0000 | 0.7614±0.7895 |
| 29 | Analyte 41 | 7.2980 | 221 | 0.3288±0.0938 | 0.3604±0.0264 | 0.4247±0.1063 | 0.4503±0.0599 |
| 30 | Analyte 42 | 7.3489 | 142 | 0.0002±0.0000 | 0.0800±0.0060 | 0.0625±0.0077 | 0.0642±0.0138 |
| 31 | pyruvic acid | 7.3839 | 174 | 0.7296±0.2028 | 0.3352±0.0273 | 0.4046±0.0930 | 0.5587±0.0816 |
| 32 | unknown | 7.4186 | 207 | 2.7897±0.7962 | 3.1423±0.1808 | 3.7971±0.8943 | 3.8386±0.6035 |
| 33 | Analyte 48 | 7.4779 | 83 | 0.2853±0.0918 | 0.3206±0.0325 | 0.4187±0.1135 | 0.4373±0.0829 |
| 34 | Analyte 49 | 7.4943 | 174 | 1.1427±0.3279 | 1.2172±0.1075 | 1.5164±0.3766 | 1.5474±0.2454 |
| 35 | lactic acid | 7.5232 | 117 | 30.3140±8.1958 | 1.1481±0.0851 | 2.3081±0.5597 | 1.9542±0.2901 |
| 36 | Analyte 52 | 7.5583 | 83 | 0.5078±0.1447 | 0.4842±0.0475 | 0.6329±0.1556 | 0.5705±0.0810 |
| 37 | Analyte 55 | 7.5938 | 85 | 0.0002±0.0000 | 0.0001±0.0000 | 0.7618±0.6722 | 0.3297±0.1745 |
| 38 | unknown | 7.6146 | 221 | 1.2158±0.3466 | 1.2079±0.0769 | 1.4819±0.3572 | 1.5251±0.2217 |
| 39 | Analyte 57 | 7.6431 | 54 | 0.0841±0.0871 | 0.2133±0.0130 | 0.1847±0.0449 | 0.2262±0.0306 |
| 40 | Analyte 58 | 7.6794 | 225 | 0.8661±0.2577 | 0.9181±0.0578 | 1.1250±0.2997 | 1.1791±0.1800 |
| 41 | glycolic acid | 7.7547 | 147 | 1.4340±0.4357 | 1.8731±0.1319 | 1.8442±0.4653 | 2.0592±0.3002 |
| 42 | Analyte 61 | 7.7707 | 258 | 0.0002±0.0000 | 0.0001±0.0000 | 0.0505±0.0066 | 0.0660±0.0030 |
| 43 | Analyte 62 | 7.7855 | 127 | 0.1113±0.0311 | 0.1311±0.0070 | 0.1179±0.0611 | 0.1818±0.0303 |
| 44 | Analyte 63 | 7.8216 | 147 | 0.1328±0.0831 | 0.2682±0.0179 | 0.2399±0.0539 | 0.2401±0.0315 |
| 45 | Analyte 64 | 7.8387 | 69 | 0.7556±0.2222 | 0.6090±0.0272 | 0.7604±0.1692 | 0.7363±0.1121 |
| 46 | Analyte 65 | 7.8998 | 57 | 0.4772±0.1372 | 0.3590±0.0168 | 0.4142±0.1050 | 0.4400±0.0596 |
| 47 | Analyte 66 | 7.9187 | 147 | 0.5995±0.2134 | 0.7079±0.0776 | 0.6387±0.1667 | 0.9460±0.1525 |
| 48 | unknown | 7.9712 | 72 | 0.0985±0.1019 | 0.1822±0.1052 | 0.1571±0.0911 | 0.8250±0.0717 |
| 49 | Analyte 72 | 7.9842 | 125 | 0.1345±0.0385 | 0.1351±0.0146 | 0.2005±0.0503 | 0.1610±0.0235 |
| 50 | unknown | 8.0373 | 174 | 0.5555±0.1679 | 0.7600±0.0701 | 0.9225±0.2357 | 0.9484±0.1394 |
| 51 | 2-keto-isovaleric acid 1 | 8.0428 | 83 | 0.0002±0.0000 | 0.0852±0.0882 | 0.2555±0.1330 | 0.3342±0.0547 |
| 52 | maleimide | 8.0559 | 154 | 0.6920±0.2242 | 0.7052±0.0604 | 0.9428±0.2463 | 1.1788±0.2118 |
| 53 | Analyte 76 | 8.0741 | 71 | 0.6251±0.1560 | 0.6228±0.0339 | 0.8245±0.1972 | 0.8884±0.1740 |
| 54 | alanine 1 | 8.1373 | 116 | 5.3949±1.7048 | 3.3496±0.2581 | 8.8926±2.2202 | 9.4598±1.3686 |
| 55 | Analyte 78 | 8.1678 | 57 | 3.7288±1.0948 | 3.7901±0.2859 | 4.9540±1.1530 | 5.2742±0.7538 |
| 56 | unknown | 8.1775 | 187 | 0.3943±0.1140 | 0.3803±0.0379 | 0.4864±0.1162 | 0.5260±0.0857 |
| 57 | Analyte 80 | 8.2020 | 174 | 0.1838±0.0541 | 0.1360±0.0712 | 0.2433±0.0643 | 0.2489±0.0395 |
| 58 | unknown | 8.2508 | 71 | 0.9241±0.2610 | 0.9320±0.0881 | 1.2044±0.2608 | 1.2951±0.2034 |
| 59 | unknown | 8.2629 | 141 | 0.9028±0.2523 | 0.8859±0.0992 | 1.1620±0.2768 | 1.0569±0.1636 |
| 60 | Analyte 84 | 8.3411 | 258 | 1.5474±0.4714 | 1.5868±0.1319 | 1.7924±0.4362 | 1.8098±0.2536 |
| 61 | Analyte 85 | 8.3614 | 71 | 0.8806±0.2338 | 0.9061±0.0749 | 1.1741±0.2793 | 1.2107±0.1694 |
| 62 | Analyte 87 | 8.4274 | 245 | 0.1061±0.0278 | 0.0879±0.0088 | 0.1192±0.0331 | 0.1187±0.0068 |
| 63 | Analyte 90 | 8.5156 | 168 | 0.2411±0.0788 | 0.2604±0.0279 | 0.2878±0.0694 | 0.3297±0.0720 |
| 64 | oxalic acid | 8.5359 | 147 | 0.2677±0.1697 | 0.5142±0.0457 | 0.4750±0.1143 | 0.6142±0.0944 |
| 65 | Analyte 93 | 8.6455 | 156 | 14.5608±4.0358 | 5.0095±0.4341 | 10.2543±2.3425 | 12.2351±1.9406 |
| 66 | 3-Hydroxypyridine | 8.6770 | 152 | 0.5384±0.1468 | 0.5410±0.0493 | 0.6671±0.1570 | 0.7836±0.1073 |
| 67 | 3-Hydroxypropionic acid 1 | 8.6846 | 147 | 16.7696±4.4852 | 15.2289±1.2696 | 22.0163±5.2193 | 17.2124±2.3712 |
| 68 | unknown | 8.7266 | 355 | 0.1056±0.0327 | 0.1168±0.0068 | 0.1325±0.0280 | 0.1272±0.0247 |
| 69 | Analyte 98 | 8.7525 | 191 | 0.2711±0.0782 | 0.2207±0.0292 | 0.2869±0.0700 | 0.1603±0.0929 |
| 70 | Analyte 99 | 8.7756 | 89 | 0.2791±0.0811 | 0.3275±0.0291 | 0.3663±0.0863 | 0.3735±0.0609 |
| 71 | 4-hydroxypyridine | 8.9093 | 152 | 0.3039±0.0919 | 0.3820±0.0280 | 0.4653±0.1100 | 0.4875±0.0685 |
| 72 | Analyte 104 | 8.9538 | 141 | 0.5443±0.1584 | 0.6009±0.0585 | 0.7658±0.1830 | 0.7904±0.1125 |
| 73 | sulfuric acid | 9.0251 | 281 | 0.5574±0.1260 | 0.8094±0.0553 | 1.4113±0.3472 | 0.8701±0.1098 |
| 74 | unknown | 9.0880 | 152 | 0.6627±0.1788 | 0.8370±0.0844 | 1.0795±0.2552 | 1.1640±0.1481 |
| 75 | Analyte 107 | 9.1614 | 207 | 0.5331±0.1134 | 0.6571±0.0619 | 0.7869±0.1866 | 0.8883±0.1063 |
| 76 | Analyte 109 | 9.2016 | 355 | 0.0273±0.0281 | 0.1053±0.0091 | 0.1280±0.0230 | 0.0476±0.0492 |
| 77 | Analyte 110 | 9.2125 | 221 | 0.4371±0.1032 | 0.4483±0.0422 | 0.5938±0.1411 | 0.5635±0.0890 |
| 78 | Analyte 112 | 9.2276 | 355 | 0.0908±0.0189 | 0.0988±0.0075 | 0.1143±0.0264 | 0.1464±0.0231 |
| 79 | α-ketoisocaproic acid 2 | 9.2545 | 110 | 0.1911±0.0404 | 0.2529±0.0237 | 0.2496±0.0519 | 0.3015±0.0379 |
| 80 | unknown | 9.3023 | 89 | 0.2627±0.0740 | 0.2382±0.0193 | 0.2760±0.0687 | 0.4112±0.0513 |
| 81 | unknown | 9.3228 | 91 | 0.1616±0.0372 | 0.3191±0.0469 | 0.1880±0.0534 | 0.5294±0.0679 |
| 82 | succinate semialdehyde 2 | 9.3693 | 113 | 0.0682±0.0122 | 0.0499±0.0096 | 0.0823±0.0220 | 0.0938±0.0185 |
| 83 | phenylacetaldehyde 2 | 9.3904 | 91 | 0.1463±0.0331 | 0.1996±0.1066 | 0.1737±0.0347 | 0.4782±0.0667 |
| 84 | Analyte 121 | 9.4466 | 221 | 0.1935±0.1101 | 0.2559±0.0316 | 0.3611±0.0844 | 0.3540±0.0517 |
| 85 | malonic acid 1 | 9.4776 | 141 | 0.1240±0.0306 | 0.1254±0.0108 | 0.1592±0.0339 | 0.1029±0.0550 |
| 86 | Analyte 123 | 9.5370 | 226 | 0.6033±0.1019 | 0.7618±0.0737 | 0.9242±0.2127 | 0.9107±0.0842 |
| 87 | unknown | 9.5776 | 281 | 0.4208±0.0609 | 0.4971±0.0483 | 0.6275±0.1404 | 0.6476±0.0863 |
| 88 | Analyte 126 | 9.6164 | 57 | 0.3886±0.0406 | 0.4009±0.0422 | 0.5419±0.1105 | 0.6083±0.0467 |
| 89 | Analyte 127 | 9.6292 | 108 | 0.0246±0.0130 | 0.0194±0.0200 | 0.0186±0.0132 | 0.0333±0.0343 |
| 90 | Analyte 128 | 9.6667 | 392 | 0.1005±0.0113 | 0.1129±0.0255 | 0.1532±0.0217 | 0.1593±0.0151 |
| 91 | valine | 9.7381 | 144 | 2.3452±0.4050 | 1.8815±0.2541 | 4.9541±1.1252 | 10.6475±0.9922 |
| 92 | Analyte 130 | 9.7893 | 57 | 0.1467±0.0808 | 0.2254±0.0210 | 0.2564±0.0465 | 0.3464±0.0193 |
| 93 | unknown | 9.8085 | 156 | 0.2253±0.0355 | 0.1902±0.0215 | 0.3069±0.0477 | 0.4523±0.0379 |
| 94 | Analyte 132 | 9.8244 | 281 | 0.0800±0.0444 | 0.1245±0.0073 | 0.1574±0.0239 | 0.1768±0.0184 |
| 95 | Analyte 133 | 9.8311 | 227 | 0.1517±0.0238 | 0.0872±0.0092 | 0.1438±0.0274 | 0.2267±0.0151 |
| 96 | Analyte 134 | 9.8644 | 230 | 0.0002±0.0000 | 0.0363±0.0193 | 0.0002±0.0000 | 0.0159±0.0163 |
| 97 | hydroxyurea | 9.9234 | 169 | 0.0389±0.0401 | 0.0485±0.0255 | 0.0800±0.0132 | 0.0836±0.0439 |
| 98 | 2-Butyne-1,4-diol | 9.9344 | 217 | 0.0912±0.0180 | 0.1560±0.0202 | 0.1624±0.0346 | 0.1493±0.0104 |
| 99 | Analyte 138 | 10.0232 | 111 | 0.1985±0.0290 | 0.2428±0.0240 | 0.2844±0.0479 | 0.3491±0.0232 |
| 100 | Analyte 139 | 10.0558 | 68 | 0.5943±0.0676 | 0.6556±0.0818 | 0.7907±0.1242 | 0.8640±0.0587 |
| 101 | Analyte 140 | 10.1276 | 108 | 0.3612±0.0491 | 0.4250±0.0588 | 0.4594±0.0752 | 0.5063±0.0407 |
| 102 | citraconic acid degr1 | 10.1754 | 71 | 0.2166±0.0184 | 0.1524±0.0215 | 0.2123±0.0198 | 0.1460±0.0756 |
| 103 | oxamic acid | 10.2066 | 171 | 0.8720±0.1139 | 0.1449±0.0169 | 0.1412±0.0416 | 0.2039±0.0207 |
| 104 | dihydroxyacetone | 10.2145 | 192 | 0.4005±0.0575 | 0.4525±0.0644 | 0.4838±0.0812 | 0.5311±0.0406 |
| 105 | Analyte 144 | 10.2243 | 83 | 0.0645±0.0667 | 0.1631±0.0245 | 0.1923±0.0189 | 0.2042±0.0276 |
| 106 | benzoic acid | 10.2559 | 179 | 0.3858±0.0554 | 0.7229±0.0930 | 1.2977±0.2121 | 0.6537±0.0609 |
| 107 | unknown | 10.3407 | 108 | 0.0841±0.0126 | 0.0808±0.0047 | 0.1048±0.0188 | 0.0966±0.0036 |
| 108 | unknown | 10.4116 | 190 | 0.0002±0.0000 | 0.0001±0.0000 | 0.0522±0.0305 | 0.0249±0.0129 |
| 109 | Analyte 149 | 10.4245 | 319 | 0.1074±0.0112 | 0.0160±0.0164 | 0.0186±0.0191 | 0.0161±0.0165 |
| 110 | ethanolamine | 10.4612 | 174 | 3.6439±0.3936 | 4.3419±0.5531 | 6.4545±0.9256 | 8.8874±0.4614 |
| 111 | phosphate | 10.5172 | 299 | 41.9272±5.2999 | 49.5016±5.0157 | 33.3330±4.8752 | 30.0440±2.0829 |
| 112 | glycerol | 10.5287 | 158 | 2.0628±0.2155 | 0.9668±0.1472 | 2.3793±0.3507 | 4.6193±2.3956 |
| 113 | Analyte 155 | 10.5836 | 108 | 0.3256±0.0379 | 0.3775±0.0415 | 0.3962±0.0449 | 0.4364±0.0260 |
| 114 | N-cyclohexylformamide 1 | 10.6576 | 71 | 0.8709±0.0726 | 0.9098±0.1290 | 1.0422±0.1062 | 1.1391±0.0561 |
| 115 | glutaraldehyde 3 | 10.7441 | 116 | 0.1675±0.0227 | 0.0857±0.0225 | 0.1962±0.0301 | 0.2390±0.0209 |
| 116 | unknown | 10.7472 | 231 | 0.0013±0.0012 | 0.0304±0.0179 | 0.0337±0.0188 | 0.0715±0.0082 |
| 117 | Analyte 162 | 10.7803 | 71 | 0.0797±0.0437 | 0.0459±0.0475 | 0.0784±0.0428 | 0.0902±0.0478 |
| 118 | 2-Deoxyerythritol | 10.8033 | 117 | 0.0381±0.0393 | 0.3222±0.0407 | 0.3946±0.0481 | 0.0002±0.0000 |
| 119 | Isoleucine | 10.8212 | 158 | 1.5505±0.1827 | 0.8749±0.1274 | 1.9336±0.2716 | 5.6270±0.1976 |
| 120 | Analyte 166 | 10.8574 | 85 | 0.0344±0.0187 | 0.0589±0.0124 | 0.0790±0.0246 | 0.0750±0.0087 |
| 121 | Analyte 168 | 10.8870 | 341 | 0.0962±0.0105 | 0.0956±0.0087 | 0.1203±0.0118 | 0.1202±0.0145 |
| 122 | Analyte 169 | 10.8958 | 180 | 0.2860±0.1514 | 0.0001±0.0000 | 0.8720±0.0998 | 0.6313±0.0302 |
| 123 | proline | 10.9033 | 142 | 1.6266±0.3668 | 0.4966±0.0393 | 1.3414±0.2918 | 15.7699±0.3856 |
| 124 | Analyte 172 | 10.9412 | 129 | 0.2308±0.0087 | 0.3175±0.0352 | 0.2913±0.0332 | 0.4215±0.0204 |
| 125 | unknown | 10.9577 | 217 | 0.1609±0.0102 | 0.2923±0.0397 | 0.1720±0.0894 | 0.3497±0.0172 |
| 126 | glycine 2 | 11.0086 | 174 | 1.1636±0.0764 | 0.4727±0.0597 | 0.7864±0.0882 | 0.8340±0.0208 |
| 127 | 1,4-Cyclohexanedione 1 | 11.0149 | 69 | 0.3814±0.0317 | 0.3371±0.0451 | 0.2307±0.1218 | 0.3906±0.0088 |
| 128 | Analyte 178 | 11.0277 | 172 | 0.0868±0.0044 | 0.0166±0.0170 | 0.0430±0.0227 | 0.0819±0.0034 |
| 129 | Analyte 179 | 11.0584 | 90 | 0.0878±0.0457 | 0.2491±0.0365 | 0.1104±0.0585 | 0.0687±0.0357 |
| 130 | unknown | 11.1176 | 204 | 0.1959±0.0098 | 0.0742±0.0768 | 0.0978±0.0528 | 0.1469±0.0764 |
| 131 | succinic acid | 11.1278 | 147 | 15.0522±0.9950 | 24.4116±2.8242 | 10.299±1.1426 | 23.3588±0.8144 |
| 132 | Analyte 183 | 11.1407 | 306 | 0.0815±0.0423 | 0.0554±0.0574 | 0.0738±0.0391 | 0.1116±0.0577 |
| 133 | Thymol | 11.1716 | 123 | 0.0763±0.0102 | 0.0928±0.0098 | 0.0973±0.0060 | 0.1066±0.0029 |
| 134 | Analyte 186 | 11.1911 | 130 | 0.1981±0.0088 | 0.0001±0.0000 | 0.1968±0.0184 | 0.2582±0.0155 |
| 135 | Analyte 187 | 11.2173 | 71 | 0.2713±0.0072 | 0.2785±0.0367 | 0.3182±0.0401 | 0.3420±0.0083 |
| 136 | Analyte 188 | 11.2476 | 69 | 0.2473±0.0142 | 0.2042±0.0171 | 0.1806±0.0162 | 0.2518±0.0202 |
| 137 | 1-Methylhydantoin 1 | 11.2938 | 71 | 1.2353±0.0389 | 1.3019±0.1571 | 1.4111±0.1183 | 1.5982±0.0322 |
| 138 | D-Glyceric acid | 11.3019 | 73 | 3.7694±0.2487 | 6.0791±0.6604 | 3.8626±0.3785 | 7.8177±0.2428 |
| 139 | unknown | 11.4250 | 71 | 0.1648±0.1209 | 0.2851±0.0407 | 0.3366±0.0398 | 0.3519±0.0244 |
| 140 | Analyte 197 | 11.4545 | 123 | 0.0547±0.0286 | 0.0949±0.0069 | 0.0879±0.0056 | 0.1164±0.0083 |
| 141 | Itaconic acid | 11.4696 | 117 | 0.2431±0.0098 | 0.2522±0.0233 | 0.3149±0.0251 | 0.6158±0.0109 |
| 142 | Analyte 199 | 11.5256 | 300 | 0.1440±0.0050 | 0.1965±0.0247 | 0.2040±0.0192 | 0.2194±0.0093 |
| 143 | Analyte 200 | 11.5371 | 71 | 0.4915±0.0261 | 0.4985±0.0550 | 0.5295±0.0353 | 0.6033±0.0146 |
| 144 | Analyte 201 | 11.5652 | 217 | 0.0781±0.0050 | 0.1515±0.0108 | 0.1316±0.0106 | 0.1489±0.0092 |
| 145 | fumaric acid | 11.6208 | 245 | 1.6817±0.0853 | 2.5246±0.2913 | 1.9300±0.1223 | 2.6478±0.1522 |
| 146 | Analyte 203 | 11.6277 | 221 | 0.0376±0.0198 | 0.0108±0.0111 | 0.0289±0.0149 | 0.0188±0.0193 |
| 147 | Analyte 204 | 11.6613 | 151 | 0.0731±0.0063 | 0.0593±0.0038 | 0.0360±0.0223 | 0.0735±0.0071 |
| 148 | 1-Indanol | 11.6919 | 57 | 2.4454±0.0501 | 2.6603±0.2880 | 2.7059±0.1667 | 2.8280±0.0510 |
| 149 | Analyte 206 | 11.7061 | 89 | 0.1732±0.0898 | 0.1914±0.0153 | 0.3243±0.0290 | 0.5193±0.0317 |
| 150 | serine 1 | 11.7178 | 204 | 4.8651±0.2035 | 3.2041±0.3880 | 5.4678±0.4377 | 8.7825±0.2226 |
| 151 | pelargonic acid | 11.7783 | 215 | 0.1121±0.0081 | 0.1013±0.0097 | 0.1091±0.0060 | 0.1150±0.0037 |
| 152 | benzyl thiocyanate | 11.8512 | 155 | 0.0729±0.0075 | 0.0717±0.0112 | 0.1284±0.0018 | 0.1990±0.0065 |
| 153 | 3-Cyanoalanine | 11.8846 | 141 | 0.6001±0.0393 | 0.3262±0.0538 | 1.8065±0.1133 | 1.3370±0.0416 |
| 154 | tartronic acid | 11.9258 | 147 | 0.4271±0.0343 | 0.3873±0.0518 | 0.1696±0.0879 | 0.5308±0.0269 |
| 155 | threonine 1 | 12.0539 | 73 | 3.2564±0.0430 | 2.3587±0.2174 | 5.3415±0.2297 | 9.6254±0.5211 |
| 156 | resorcinol | 12.0605 | 217 | 0.1190±0.0621 | 0.2683±0.0168 | 0.2263±0.1181 | 0.4004±0.0288 |
| 157 | Analyte 221 | 12.0977 | 116 | 0.0274±0.0282 | 0.0544±0.0286 | 0.0324±0.0334 | 0.4309±0.0332 |
| 158 | Analyte 223 | 12.1689 | 174 | 0.0909±0.0098 | 0.0848±0.0082 | 0.1054±0.0126 | 0.0815±0.0037 |
| 159 | glutaric Acid | 12.2209 | 147 | 1.0266±0.1089 | 0.8147±0.4229 | 0.8447±0.1289 | 2.0130±0.1531 |
| 160 | Analyte 226 | 12.2370 | 305 | 0.1079±0.0071 | 0.0890±0.0071 | 0.1023±0.0058 | 0.1085±0.0170 |
| 161 | Analyte 228 | 12.2547 | 204 | 0.3188±0.0866 | 0.2827±0.0153 | 0.2951±0.0451 | 0.1750±0.0960 |
| 162 | Analyte 232 | 12.3471 | 57 | 0.1703±0.0094 | 0.1377±0.0132 | 0.1053±0.0545 | 0.0747±0.0403 |
| 163 | Analyte 234 | 12.3682 | 373 | 0.1615±0.0076 | 0.1685±0.0080 | 0.1323±0.0052 | 0.0675±0.0365 |
| 164 | methyl trans-cinnamate | 12.4018 | 103 | 0.2918±0.0191 | 0.0001±0.0000 | 0.0002±0.0000 | 0.0002±0.0000 |
| 165 | N-Ethylglycine 1 | 12.4208 | 174 | 0.6096±0.0249 | 0.6292±0.0304 | 0.6683±0.0114 | 0.6767±0.0328 |
| 166 | unknown | 12.5532 | 174 | 1.1372±0.0704 | 1.1816±0.0773 | 1.2474±0.0394 | 1.2280±0.0811 |
| 167 | unknown | 12.5893 | 160 | 0.1450±0.0755 | 0.3389±0.0257 | 0.4095±0.0360 | 0.3969±0.0121 |
| 168 | unknown | 12.6380 | 248 | 0.0485±0.0129 | 0.0801±0.0104 | 0.0782±0.0080 | 0.1053±0.0004 |
| 169 | β-Alanine 2 | 12.6769 | 174 | 0.7013±0.0558 | 0.7393±0.0456 | 0.7825±0.0230 | 0.7479±0.0436 |
| 170 | 3-Aminoisobutyric acid 1 | 13.0377 | 174 | 1.1575±0.1037 | 1.2407±0.0565 | 1.3648±0.0782 | 1.2357±0.0564 |
| 171 | Analyte 249 | 13.2668 | 73 | 0.0002±0.0000 | 0.7545±0.0517 | 1.0743±0.0886 | 0.9457±0.1047 |
| 172 | unknown | 13.4152 | 283 | 0.1855±0.0962 | 0.0001±0.0000 | 0.1583±0.0099 | 0.0002±0.0000 |
| 173 | L-Malic acid | 13.4267 | 73 | 723.0736±41.5075 | 549.5456±26.9075 | 383.9078±17.9641 | 502.8391±32.7151 |
| 174 | Analyte 252 | 13.4712 | 305 | 0.2597±0.0038 | 0.2626±0.0077 | 0.1882±0.0185 | 0.2406±0.0072 |
| 175 | Analyte 253 | 13.4836 | 71 | 0.6685±0.0233 | 0.5683±0.0329 | 0.5687±0.0277 | 0.5784±0.0201 |
| 176 | Analyte 255 | 13.6103 | 73 | 2.9670±0.0741 | 3.4880±0.1887 | 5.1478±0.3877 | 4.7608±0.3447 |
| 177 | asparagine 4 | 13.6229 | 115 | 0.7416±0.0404 | 0.2656±0.0444 | 1.6015±0.0657 | 1.3817±0.0677 |
| 178 | 4-acetamidobutyric acid 2 | 13.6468 | 174 | 0.4240±0.0270 | 0.6119±0.0375 | 0.5808±0.0330 | 0.5834±0.0239 |
| 179 | 1,5-anhydroglucitol | 13.6911 | 57 | 0.3496±0.0334 | 0.3818±0.0248 | 0.3787±0.0297 | 0.3907±0.0371 |
| 180 | aspartic acid 1 | 13.7954 | 232 | 2.6165±0.1261 | 3.6312±0.2420 | 5.2833±0.3081 | 7.0189±0.1906 |
| 181 | oxoproline | 13.8593 | 156 | 56.2631±2.0622 | 55.7584±4.0728 | 93.1578±4.3214 | 181.2674±5.3303 |
| 182 | 4-aminobutyric acid 1 | 13.9367 | 174 | 3.5629±0.1753 | 7.9191±0.3968 | 6.7721±0.3502 | 7.2658±0.1358 |
| 183 | Analyte 264 | 13.9461 | 314 | 0.0353±0.0365 | 0.0318±0.0286 | 0.2483±0.0244 | 0.2085±0.0072 |
| 184 | Analyte 265 | 13.9690 | 447 | 0.1570±0.0136 | 0.1900±0.0167 | 0.1533±0.0071 | 0.1711±0.0126 |
| 185 | Analyte 267 | 14.0025 | 185 | 0.0002±0.0000 | 0.0561±0.0307 | 0.1024±0.0124 | 0.1085±0.0563 |
| 186 | unknown | 14.0373 | 73 | 1.8233±0.0834 | 3.0152±0.0371 | 1.5780±0.0730 | 1.9413±0.1427 |
| 187 | glutamine 3 | 14.0455 | 57 | 1.2774±0.1914 | 1.3557±0.1323 | 1.3184±0.0610 | 1.3732±0.0242 |
| 188 | Analyte 270 | 14.0759 | 263 | 0.2938±0.0146 | 0.3018±0.0192 | 0.3846±0.0157 | 0.3991±0.0180 |
| 189 | unknown | 14.1043 | 156 | 0.4350±0.0254 | 0.3756±0.0190 | 0.3490±0.1857 | 0.9352±0.0418 |
| 190 | maleamate 1 | 14.1668 | 55 | 0.5116±0.0229 | 0.1780±0.0043 | 0.2734±0.0197 | 0.3104±0.0208 |
| 191 | unknown | 14.1994 | 120 | 0.2641±0.0045 | 0.0873±0.0452 | 0.3391±0.0160 | 0.5162±0.0310 |
| 192 | threonic acid | 14.2441 | 73 | 71.8376±1.9247 | 37.9539±1.2862 | 17.5380±0.6613 | 43.8282±1.4866 |
| 193 | Analyte 275 | 14.2879 | 71 | 0.1669±0.0154 | 0.1737±0.0025 | 0.1634±0.0143 | 0.2018±0.0120 |
| 194 | phenylethylamine | 14.4078 | 174 | 0.7874±0.0479 | 0.0001±0.0000 | 0.1380±0.0059 | 0.6063±0.0258 |
| 195 | α-ketoglutaric acid | 14.4709 | 73 | 42.0559±1.7193 | 10.4478±0.1971 | 20.5295±0.4345 | 20.6019±0.4602 |
| 196 | Analyte 283 | 14.5333 | 117 | 0.0006±0.0004 | 0.1817±0.0018 | 0.2233±0.0116 | 0.1456±0.0124 |
| 197 | Analyte 284 | 14.6014 | 217 | 0.1717±0.0100 | 0.3855±0.0014 | 0.2895±0.0200 | 0.3048±0.0194 |
| 198 | unknown | 14.6202 | 133 | 1.1760±0.0426 | 1.5204±0.0185 | 1.4953±0.0776 | 1.5651±0.1152 |
| 199 | D-erythronolactone 2 | 14.7215 | 117 | 3.0945±0.1280 | 2.2819±0.0274 | 1.4214±0.0948 | 3.0792±0.1750 |
| 200 | Analyte 289 | 14.7517 | 217 | 0.3544±0.0278 | 0.5628±0.2326 | 0.2586±0.2680 | 0.5077±0.2903 |
| 201 | digitoxose 2 | 14.8329 | 204 | 0.4591±0.0236 | 0.4942±0.0080 | 0.3977±0.0230 | 0.4037±0.0238 |
| 202 | Analyte 291 | 14.8698 | 217 | 0.1271±0.0658 | 1.4212±0.0611 | 1.2602±0.0505 | 1.3548±0.0907 |
| 203 | Analyte 292 | 14.9226 | 73 | 3.5804±0.0069 | 5.2528±0.1090 | 4.6197±0.1021 | 3.6547±0.2689 |
| 204 | glutamic acid | 14.9878 | 246 | 0.5488±0.0304 | 0.2798±0.0071 | 0.6062±0.0537 | 0.7310±0.0184 |
| 205 | Analyte 296 | 15.0099 | 275 | 0.1081±0.0093 | 0.0926±0.0042 | 0.1183±0.0084 | 0.0958±0.0010 |
| 206 | phenylalanine 1 | 15.0975 | 218 | 1.8800±0.1059 | 0.7463±0.0207 | 2.4564±0.2154 | 3.9858±0.1076 |
| 207 | fluorene | 15.1181 | 229 | 0.3520±0.0365 | 0.1218±0.0636 | 1.2860±0.0752 | 0.6897±0.0173 |
| 208 | creatine degr | 15.1769 | 73 | 151.5485±2.2730 | 209.5254±6.6584 | 336.7342±8.8550 | 257.9726±13.6428 |
| 209 | allose 1 | 15.3140 | 201 | 0.4758±0.0372 | 0.6792±0.0124 | 0.7032±0.0076 | 0.7174±0.0248 |
| 210 | xylose 1 | 15.3813 | 103 | 42.5176±1.2046 | 95.3002±0.3439 | 128.3749±1.2930 | 121.8544±3.7498 |
| 211 | ribose | 15.4577 | 103 | 38.4793±1.0562 | 97.8114±0.9879 | 102.7404±1.1703 | 94.1446±2.8718 |
| 212 | Analyte 307 | 15.5182 | 67 | 0.2505±0.0163 | 0.1271±0.0035 | 0.1661±0.0109 | 0.1345±0.0154 |
| 213 | unknown | 15.5723 | 116 | 0.4164±0.0295 | 0.1203±0.0624 | 1.3921±0.0131 | 1.2016±0.0251 |
| 214 | unknown | 15.6019 | 205 | 0.3643±0.1915 | 0.8938±0.0280 | 0.2841±0.2945 | 0.6144±0.3193 |
| 215 | unknown | 15.6135 | 103 | 3.6803±0.1051 | 3.7998±0.0853 | 4.6836±0.1208 | 5.0291±0.0102 |
| 216 | unknown | 15.6199 | 231 | 0.2447±0.1278 | 1.2890±0.6712 | 0.1382±0.1315 | 0.0134±0.0137 |
| 217 | ribonic acid, γ-lactone | 15.6901 | 73 | 4.8974±4.0908 | 2.0930±0.0281 | 1.3423±0.0571 | 1.5290±0.0508 |
| 218 | xylitol | 15.7596 | 73 | 0.0002±0.0000 | 0.2474±0.1282 | 0.2152±0.2230 | 0.2792±0.1448 |
| 219 | Analyte 316 | 15.8027 | 117 | 0.0002±0.0000 | 0.1084±0.0060 | 0.0528±0.0545 | 0.0759±0.0392 |
| 220 | unknown | 15.8422 | 231 | 0.0938±0.0025 | 0.2698±0.0173 | 0.2985±0.0094 | 0.2281±0.0070 |
| 221 | Levoglucosan | 15.9374 | 204 | 0.2378±0.0114 | 0.5044±0.0092 | 0.4559±0.0203 | 0.4915±0.0321 |
| 222 | Analyte 320 | 15.9563 | 255 | 0.0002±0.0000 | 0.0197±0.0203 | 0.0002±0.0000 | 0.0413±0.0214 |
| 223 | unknown | 15.9926 | 71 | 0.2588±0.0077 | 0.2371±0.0022 | 0.2698±0.0214 | 0.2606±0.0197 |
| 224 | unknown | 16.0817 | 154 | 0.0402±0.0415 | 0.3011±0.0126 | 0.0651±0.0673 | 0.0688±0.0712 |
| 225 | diglycerol 2 | 16.1528 | 205 | 6.4454±0.1897 | 7.6729±0.1056 | 6.5274±0.0952 | 6.8544±0.1294 |
| 226 | 6-deoxy-D-glucose 2 | 16.2932 | 86 | 0.0002±0.0000 | 0.1984±0.0059 | 0.1106±0.0036 | 0.0515±0.0266 |
| 227 | fucose 2 | 16.3102 | 117 | 0.0002±0.0000 | 0.6640±0.0279 | 0.3889±0.2226 | 0.0002±0.0000 |
| 228 | unknown | 16.4630 | 73 | 4.1152±0.0512 | 1.4700±0.0970 | 1.3553±0.1095 | 1.3732±0.0908 |
| 229 | Analyte 333 | 16.4767 | 57 | 0.6920±0.0373 | 0.5934±0.0202 | 0.6770±0.0132 | 0.6337±0.0347 |
| 230 | glucose-1-phosphate | 16.5428 | 217 | 0.5210±0.2704 | 2.5613±0.0426 | 1.6122±0.0241 | 1.4215±0.0417 |
| 231 | uracil-5-carboxylic acid | 16.5636 | 357 | 0.2772±0.0150 | 0.1236±0.0085 | 0.1069±0.0565 | 0.0677±0.0700 |
| 232 | 3,6-Anhydro-D-galactose 4 | 16.5789 | 292 | 0.2514±0.1305 | 0.6171±0.0205 | 0.3562±0.0337 | 0.3768±0.0171 |
| 233 | 2-deoxy-D-glucose 2 | 16.6380 | 217 | 4.7662±0.2921 | 11.1438±0.1081 | 10.0646±0.3306 | 6.4736±0.2142 |
| 234 | 4-hydroxy-3-methoxybenzoic acid; | 16.6510 | 297 | 0.0514±0.0268 | 0.0887±0.0064 | 0.0748±0.0389 | 0.0800±0.0435 |
| 235 | farnesal 2 | 16.6757 | 226 | 1.8380±0.0603 | 3.5287±0.0339 | 3.4369±0.1513 | 3.3178±0.0966 |
| 236 | 2-Deoxy-D-galactose 2 | 16.7064 | 257 | 0.1969±0.0064 | 0.1016±0.0526 | 0.0499±0.0515 | 0.1491±0.0157 |
| 237 | unknown | 16.7751 | 217 | 0.2684±0.1396 | 0.7017±0.0152 | 0.5548±0.1294 | 0.8323±0.2522 |
| 238 | Analyte 347 | 16.9370 | 219 | 0.0002±0.0000 | 0.0765±0.0029 | 0.0200±0.0205 | 0.0002±0.0000 |
| 239 | Analyte 348 | 17.0432 | 117 | 0.0002±0.0000 | 2.1251±0.0254 | 0.3870±0.2008 | 0.5603±0.0294 |
| 240 | Analyte 350 | 17.1064 | 204 | 5.6199±0.2071 | 2.3648±0.0698 | 2.4858±0.0413 | 1.4304±0.0585 |
| 241 | citric acid | 17.1966 | 273 | 7.7670±0.1178 | 1.1233±0.0325 | 1.6421±0.0585 | 1.7693±0.0512 |
| 242 | α-D-glucosamine 1-phosphate | 17.1732 | 103 | 17.7074±0.7362 | 0.3160±0.3275 | 0.3015±0.3125 | 0.0002±0.0000 |
| 243 | unknown | 17.3628 | 73 | 0.0002±0.0000 | 0.6029±0.3193 | 0.9504±0.0522 | 0.0501±0.0517 |
| 244 | tagatose 1 | 17.4613 | 157 | 0.9136±0.0637 | 0.2416±0.1909 | 0.1767±0.1830 | 0.2894±0.1594 |
| 245 | sorbose 2 | 17.8210 | 307 | 392.4361±203.5146 | 341.4442±186.0861 | 543.1414±281.7888 | 675.8267±85.6478 |
| 246 | gluconic lactone 2 | 17.9980 | 308 | 28.5356±1.0958 | 198.3875±42.5335 | 332.3491±54.5817 | 244.5360±30.1527 |
| 247 | mannose 2 | 18.0639 | 409 | 0.9061±0.0702 | 0.5710±0.0654 | 0.3056±0.0263 | 0.7245±0.0863 |
| 248 | galactose 2 | 18.1510 | 389 | 1.2749±0.0586 | 1.2772±0.1913 | 1.6571±0.1810 | 1.8587±0.2134 |
| 249 | glucose 2 | 18.2860 | 160 | 140.4215±8.8771 | 144.3141±22.1800 | 160.6146±18.1407 | 215.1765±27.8294 |
| 250 | octanal 3 | 18.3585 | 304 | 0.0002±0.0000 | 0.0286±0.0295 | 0.0964±0.0549 | 0.1414±0.0751 |
| 251 | glucuronic acid 2 | 18.3956 | 159 | 1.8460±0.1251 | 1.4918±0.2204 | 2.1517±0.2356 | 2.9630±0.4400 |
| 252 | D-galacturonic acid 1 | 18.4104 | 333 | 0.3696±0.0300 | 0.5017±0.0754 | 0.9938±0.1073 | 1.6778±0.2265 |
| 253 | sorbitol | 18.4543 | 159 | 1.9220±0.1285 | 1.2655±0.1871 | 1.8277±0.2014 | 2.5326±0.3751 |
| 254 | tyrosine 1 | 18.4955 | 218 | 0.8262±0.0599 | 0.7040±0.1234 | 1.6534±0.1110 | 2.4314±0.3119 |
| 255 | unknown | 18.5362 | 333 | 0.0002±0.0000 | 0.0865±0.0896 | 0.0002±0.0000 | 0.2478±0.0346 |
| 256 | 4-hydroxycinnamic acid | 18.5526 | 293 | 0.6909±0.0574 | 0.9661±0.1583 | 1.4255±0.1766 | 0.9325±0.1749 |
| 257 | unknown | 18.5639 | 244 | 0.3742±0.0329 | 0.3780±0.0537 | 0.6349±0.0757 | 0.6013±0.0879 |
| 258 | conduritol b epoxide 2 | 18.6147 | 361 | 0.4632±0.0453 | 0.5133±0.0839 | 0.6543±0.0732 | 0.8302±0.0877 |
| 259 | unknown | 18.6773 | 292 | 0.3075±0.0385 | 0.1949±0.0302 | 0.1670±0.0871 | 0.2083±0.0306 |
| 260 | unknown | 18.7025 | 57 | 0.8825±0.0411 | 0.5863±0.0920 | 1.1615±0.1107 | 1.0988±0.1886 |
| 261 | Analyte 384 | 18.7736 | 143 | 0.0002±0.0000 | 0.2945±0.0480 | 0.4413±0.0465 | 0.4693±0.0748 |
| 262 | Analyte 385 | 18.7832 | 204 | 280.3506±18.7540 | 1.8879±0.2213 | 2.6066±0.5181 | 2.4115±0.2481 |
| 263 | Analyte 388 | 18.8664 | 71 | 0.1469±0.0074 | 0.0980±0.0221 | 0.0619±0.0640 | 0.1700±0.0323 |
| 264 | galactonic acid | 18.9697 | 292 | 1.7055±0.1102 | 1.5301±0.2264 | 1.9072±0.1869 | 1.1620±0.1585 |
| 265 | guanine 2 | 19.0753 | 324 | 0.0002±0.0000 | 0.1735±0.0348 | 0.3602±0.0361 | 0.2682±0.0404 |
| 266 | saccharic acid | 19.1368 | 333 | 25.2728±1.6090 | 14.3351±2.2928 | 23.1925±2.4832 | 18.7993±2.7243 |
| 267 | unknown | 19.2017 | 305 | 0.1459±0.0101 | 0.1174±0.0271 | 0.1547±0.0230 | 0.1442±0.0187 |
| 268 | Analyte 399 | 19.2622 | 204 | 1.4670±0.0889 | 1.8128±0.2962 | 2.1459±0.2337 | 2.7374±0.3832 |
| 269 | unknown | 19.2700 | 318 | 0.4520±0.0198 | 0.4745±0.0791 | 0.6741±0.0660 | 0.5266±0.0766 |
| 270 | 4-Hydroxymethyl-3-methoxyphenoxyacetic acid | 19.3326 | 324 | 0.0269±0.0277 | 0.2821±0.0402 | 0.5774±0.0571 | 0.4299±0.0640 |
| 271 | xanthine | 19.3357 | 292 | 0.0002±0.0000 | 0.0001±0.0000 | 0.0647±0.0336 | 0.0002±0.0000 |
| 272 | unknown | 19.4157 | 378 | 0.1689±0.0194 | 0.0801±0.0180 | 0.2496±0.0226 | 0.3357±0.0445 |
| 273 | mucic acid | 19.4196 | 333 | 0.0829±0.0439 | 0.0602±0.0313 | 0.1521±0.0795 | 0.1199±0.0640 |
| 274 | Analyte 405 | 19.4783 | 200 | 0.1410±0.0196 | 0.1827±0.0308 | 0.2669±0.0229 | 0.2793±0.0420 |
| 275 | palmitic acid | 19.5374 | 117 | 19.0647±1.2403 | 11.2463±1.7919 | 21.1096±2.2815 | 20.7198±3.0838 |
| 276 | unknown | 19.5511 | 217 | 0.1418±0.1468 | 0.3012±0.0520 | 0.4690±0.0517 | 0.0002±0.0000 |
| 277 | L-dopa 1 | 19.6172 | 267 | 0.3128±0.0577 | 0.7597±0.0864 | 0.6077±0.0919 | 0.8294±0.1331 |
| 278 | unknown | 19.7690 | 73 | 0.3007±0.1728 | 0.7899±0.1568 | 1.1271±0.0791 | 1.0183±0.2330 |
| 279 | myo-inositol | 19.9229 | 217 | 219.6579±14.2465 | 137.8668±21.8557 | 487.2160±54.7568 | 247.3408±34.4356 |
| 280 | ferulic acid | 19.9708 | 338 | 0.0501±0.0036 | 0.0604±0.0219 | 0.0717±0.0085 | 0.1166±0.0156 |
| 281 | unknown | 20.0231 | 245 | 0.2951±0.0137 | 0.0453±0.0469 | 0.0002±0.0000 | 0.1312±0.0312 |
| 282 | N-Acetyl-β-D-mannosamine 4 | 20.0320 | 205 | 0.5547±0.0421 | 0.9219±0.1495 | 0.8361±0.0765 | 0.6206±0.0927 |
| 283 | Analyte 417 | 20.1360 | 290 | 0.0919±0.0137 | 0.0989±0.0194 | 0.2486±0.0312 | 0.0931±0.0487 |
| 284 | unknown | 20.1823 | 217 | 0.4070±0.0118 | 0.2309±0.0293 | 0.4452±0.0640 | 0.0450±0.0465 |
| 285 | Analyte 420 | 20.1969 | 174 | 0.3866±0.0177 | 0.0001±0.0000 | 0.0414±0.0428 | 0.1334±0.0706 |
| 286 | unknown | 20.2445 | 319 | 0.3052±0.0224 | 0.6305±0.0954 | 0.8775±0.1070 | 0.5169±0.0952 |
| 287 | d-Glucoheptose 1 | 20.3025 | 319 | 0.3866±0.0325 | 0.8647±0.1242 | 1.2154±0.1157 | 0.6698±0.1391 |
| 288 | caffeic acid | 20.3588 | 219 | 1.3195±0.2397 | 2.7767±0.4328 | 2.2184±0.2677 | 3.9901±0.6824 |
| 289 | N-methyltryptophan | 20.4427 | 117 | 0.0002±0.0000 | 0.0253±0.0132 | 0.0002±0.0000 | 0.0234±0.0240 |
| 290 | Analyte 433 | 20.5287 | 361 | 0.0430±0.0095 | 0.2784±0.0461 | 0.0628±0.0097 | 0.4635±0.0680 |
| 291 | noradrenaline | 20.5433 | 174 | 0.2784 ±0.0180 | 0.2473±0.0342 | 0.3102±0.0386 | 0.5333±0.0689 |
| 292 | phytol | 20.6485 | 71 | 0.4035 ±0.0192 | 0.2842±0.0474 | 0.5452±0.0587 | 0.5050±0.0786 |
| 293 | Analyte 437 | 20.7084 | 204 | 0.2373 ±0.0330 | 0.1641±0.0206 | 0.3280±0.0559 | 0.5078±0.0580 |
| 294 | β-Mannosylglycerate 1 | 20.9344 | 217 | 0.1228 ±0.0160 | 0.3455±0.0451 | 0.3756±0.0252 | 0.2923±0.0501 |
| 295 | fructose 2,6-biphosphate degr prod 2 | 20.9696 | 211 | 0.2162 ±0.0090 | 0.0476±0.0251 | 0.1259±0.0089 | 0.1234±0.0163 |
| 296 | tryptophan 1 | 21.0448 | 202 | 0.3561 ±0.0169 | 1.2833±0.1868 | 1.5376±0.1698 | 1.5646±0.2058 |
| 297 | linolenic acid | 21.0996 | 75 | 0.6032 ±0.0326 | 0.3715±0.0792 | 0.5610±0.0499 | 0.6771±0.0808 |
| 298 | elaidic acid | 21.2557 | 131 | 0.3851 ±0.0155 | 0.2452±0.0392 | 0.4903±0.0505 | 0.4477±0.0645 |
| 299 | stearic acid | 21.3278 | 117 | 8.5664 ±0.6027 | 5.1139±0.8192 | 9.8044±1.1055 | 9.7234±1.4430 |
| 300 | unknown | 21.4017 | 204 | 0.5002 ±0.0441 | 0.0521±0.0063 | 0.5927±0.0695 | 0.2297±0.0926 |
| 301 | glucoheptonic acid 1 | 21.5357 | 73 | 4.4175 ±0.2151 | 4.9083±0.7271 | 7.5043±0.7802 | 5.3784±0.7920 |
| 302 | Analyte 452 | 21.6684 | 73 | 3.7055 ±0.2849 | 2.2424±0.3118 | 3.1586±0.3788 | 3.2669±0.2538 |
| 303 | Phenyl β-D-glucopyranoside | 21.7007 | 306 | 0.0002 ±0.0000 | 0.0832±0.0458 | 0.0322±0.0332 | 0.0427±0.0441 |
| 304 | Analyte 455 | 21.7570 | 159 | 0.0857 ±0.0452 | 0.0944±0.0212 | 1.0229±0.1108 | 0.4375±0.0658 |
| 305 | unknown | 21.7794 | 387 | 0.1605 ±0.0127 | 0.0849±0.0084 | 0.1371±0.0172 | 0.1335±0.0194 |
| 306 | Analyte 457 | 21.8272 | 204 | 1.8930 ±0.1390 | 2.7418±0.4225 | 3.3383±0.3638 | 3.6157±0.5281 |
| 307 | glucose-6-phosphate 1 | 21.8749 | 387 | 0.4698 ±0.0276 | 0.1972±0.0228 | 0.3310±0.0454 | 0.2693±0.0303 |
| 308 | Analyte 459 | 21.9285 | 204 | 0.0480 ±0.0248 | 0.0454±0.0255 | 0.4505±0.0452 | 0.1558±0.0294 |
| 309 | unknown | 22.0417 | 387 | 0.1176±0.0200 | 0.0481±0.0107 | 0.0763±0.0096 | 0.0690±0.0070 |
| 310 | Analyte 466 | 22.1944 | 73 | 4.1612 ±0.3948 | 1.1360±0.1827 | 4.5385±0.5082 | 1.6873±0.2095 |
| 311 | Analyte 467 | 22.2224 | 73 | 4.1736±0.2950 | 4.1376±0.6294 | 1.5037±0.1522 | 3.4954±0.4739 |
| 312 | purine riboside | 22.3357 | 204 | 0.6029±0.0573 | 0.3893±0.0579 | 3.9253±0.4567 | 2.1283±0.2799 |
| 313 | Analyte 470 | 22.4557 | 57 | 0.1372±0.1421 | 0.1345±0.0736 | 0.3014±0.1635 | 0.3097±0.1914 |
| 314 | Analyte 471 | 22.4695 | 91 | 3.9095±0.2799 | 2.4024±0.3752 | 3.8144±0.4208 | 2.6690±0.3676 |
| 315 | Analyte 474 | 22.6101 | 204 | 0.2397±0.0145 | 0.1764±0.0360 | 2.8274±0.3166 | 0.4101±0.0599 |
| 316 | 6-phosphogluconic acid | 22.6854 | 204 | 0.5294±0.0354 | 0.3823±0.0514 | 0.9117±0.1100 | 0.6142±0.0708 |
| 317 | Analyte 477 | 22.7868 | 73 | 0.8519±0.0101 | 0.7883±0.1554 | 0.6772±0.0648 | 0.8590±0.1000 |
| 318 | Analyte 479 | 22.8836 | 105 | 1.2034±0.0743 | 1.3023±0.2044 | 4.8358±0.5291 | 1.2702±0.1342 |
| 319 | Analyte 480 | 22.9716 | 105 | 0.0761±0.0402 | 0.0867±0.0485 | 0.4356±0.0452 | 0.4565±0.0436 |
| 320 | Arachidic acid | 22.9829 | 117 | 0.0002±0.0000 | 0.0835±0.0174 | 0.1847±0.0231 | 0.2012±0.0358 |
| 321 | Analyte 486 | 23.1734 | 446 | 0.5991±0.0356 | 0.0626±0.0087 | 0.0473±0.0245 | 0.4434±0.0555 |
| 322 | Analyte 487 | 23.1997 | 204 | 0.0002±0.0000 | 0.5142±0.0867 | 1.0491±0.1277 | 0.3998±0.0521 |
| 323 | neohesperidin | 23.2524 | 363 | 0.1786±0.0174 | 0.0950±0.0100 | 0.2015±0.0204 | 0.1149±0.0116 |
| 324 | DL-dihydrosphingosine 1 | 23.3274 | 204 | 3.6211±0.2422 | 2.5356±0.3847 | 8.7831±1.0230 | 3.3603±0.4189 |
| 325 | Analyte 491 | 23.3781 | 446 | 0.9245±0.0590 | 0.0738±0.0096 | 0.0663±0.0146 | 0.5940±0.0656 |
| 326 | Analyte 493 | 23.4600 | 55 | 1.5541±0.0969 | 0.5277±0.0438 | 0.3876±0.0339 | 0.2541±0.1359 |
| 327 | Analyte 497 | 23.6795 | 268 | 0.0394±0.0170 | 0.0172±0.0092 | 0.1373±0.0166 | 0.9775±0.1359 |
| 328 | arbutin | 23.8561 | 217 | 0.1693±0.0436 | 0.1855±0.0341 | 0.0598±0.0618 | 0.3438±0.0660 |
| 329 | Analyte 501 | 23.8733 | 204 | 0.0002±0.0000 | 0.0357±0.0369 | 0.3289±0.0415 | 0.0887±0.0251 |
| 330 | Analyte 505 | 24.1992 | 217 | 0.9265±0.0991 | 0.0001±0.0000 | 2.4020±0.2892 | 1.8536±0.2313 |
| 331 | unknown | 24.4749 | 437 | 78.2741±40.5991 | 89.3237±14.5529 | 138.4900±12.3124 | 142.7182±11.7145 |
| 332 | Analyte 511 | 24.5976 | 362 | 0.0002±0.0000 | 2177.4598±1131.5064 | 2322.5593±245.9883 | 1648.0735±224.1298 |
| 333 | Analyte 512 | 24.6091 | 179 | 0.0002±0.0000 | 12.1114±1.9236 | 17.1139±1.6975 | 11.1452±1.4610 |
| 334 | lactose 2 | 24.7758 | 307 | 0.0837±0.0077 | 0.2607±0.0392 | 0.3181±0.0322 | 0.3120±0.0366 |
| 335 | unknown | 24.8948 | 307 | 0.1465±0.0230 | 0.4117±0.0628 | 0.4649±0.0436 | 0.4805±0.0778 |
| 336 | lactulose 1 | 24.9027 | 319 | 0.0786±0.0440 | 0.2849±0.1491 | 0.3414±0.0337 | 0.3533±0.0575 |
| 337 | unknown | 25.0088 | 480 | 0.0002±0.0000 | 0.0530±0.0283 | 0.0310±0.0320 | 0.0685±0.0410 |
| 338 | unknown | 25.0189 | 217 | 0.9469±0.0760 | 2.2691±0.3295 | 2.5359±0.2104 | 2.9334±0.4199 |
| 339 | unknown | 25.0649 | 370 | 0.1201±0.0004 | 0.1287±0.0215 | 0.2413±0.0269 | 0.1156±0.0218 |
| 340 | 11-β-prostaglandin-F-2-α 1 | 25.0790 | 193 | 0.6173±0.0424 | 1.5208±0.2191 | 3.2410±0.3656 | 0.9607±0.1471 |
| 341 | cellobiose 2 | 25.1101 | 204 | 0.5289±0.2819 | 1.0061±0.1303 | 3.2190±0.3480 | 1.7880±0.2871 |
| 342 | trehalose | 25.2259 | 191 | 0.2335±0.0305 | 0.1055±0.0555 | 0.2921±0.0298 | 0.2660±0.0449 |
| 343 | unknown | 25.2325 | 204 | 0.3971±0.0409 | 0.6691±0.0982 | 1.1398±0.1408 | 0.9485±0.1249 |
| 344 | maltose | 25.2801 | 160 | 0.2272±0.0330 | 0.5531±0.1011 | 0.6467±0.0744 | 0.6851±0.0883 |
| 345 | lactobionic Acid 1 | 25.3577 | 193 | 0.3770±0.0320 | 0.0961±0.0293 | 6.6445±0.7287 | 0.9417±0.1146 |
| 346 | unknown | 25.3659 | 361 | 0.2233±0.0350 | 0.2206±0.0263 | 0.3424±0.1580 | 0.2239±0.1210 |
| 347 | sophorose 1 | 25.4287 | 319 | 0.1339±0.0035 | 0.1428±0.0215 | 0.6825±0.0795 | 0.4429±0.0544 |
| 348 | lactitol | 25.4824 | 191 | 0.8294±0.0545 | 0.9363±0.1417 | 1.1315±0.0957 | 0.7410±0.1264 |
| 349 | unknown | 25.6816 | 204 | 0.2026±0.0348 | 0.5045±0.0681 | 0.7956±0.0983 | 0.7329±0.1067 |
| 350 | Gentiobiose 1 | 25.7433 | 204 | 0.3762±0.0177 | 0.9493±0.1526 | 1.1258±0.1149 | 1.2092±0.1777 |
| 351 | unknown | 25.8055 | 103 | 0.0338±0.0349 | 0.3898±0.0881 | 0.5049±0.0260 | 0.4444±0.0287 |
| 352 | melibiose 1 | 26.0543 | 204 | 0.5468±0.0318 | 0.9443±0.1525 | 0.9415±0.1052 | 1.3737±0.1865 |
| 353 | unknown | 26.1201 | 204 | 0.3088±0.0209 | 0.4325±0.0792 | 0.6206±0.0742 | 0.5503±0.3101 |
| 354 | unknown | 26.2452 | 204 | 0.3000±0.0150 | 0.2184±0.0562 | 0.4031±0.0393 | 0.1249±0.1293 |
| 355 | unknown | 26.4244 | 73 | 0.4867±0.0369 | 0.2887±0.0211 | 0.9688±0.1135 | 0.3075±0.0370 |
| 356 | unknown | 26.5508 | 297 | 0.2199±0.0090 | 0.2695±0.0472 | 0.5467±0.0534 | 0.5995±0.0696 |
| 357 | unknown | 26.5991 | 204 | 0.9698±0.0981 | 0.7742±0.2396 | 0.6739±0.3592 | 0.6721±0.1046 |
| 358 | Analyte 555 | 26.7778 | 204 | 0.2783±0.0242 | 0.2272±0.0383 | 0.4152±0.0674 | 0.2754±0.0283 |
| 359 | Galactinol 1 | 26.8920 | 204 | 0.5306±0.0830 | 5.1398±0.7979 | 0.5781±0.0666 | 3.1864±0.4466 |
| 360 | unknown | 26.9920 | 204 | 4.9139±0.3048 | 3.9231±0.6358 | 6.6078±0.6098 | 4.6698±0.6785 |
| 361 | Analyte 560 | 27.1033 | 204 | 0.0002±0.0000 | 0.2438±0.0427 | 0.0360±0.0371 | 0.1580±0.0068 |
| 362 | Analyte 568 | 27.6059 | 219 | 0.0779±0.0129 | 0.1790±0.0280 | 0.3310±0.0397 | 0.1817±0.0298 |
| 363 | chlorogenic acid 1 | 27.7715 | 219 | 1.2531±0.0722 | 0.6744±0.0662 | 1.5069±0.1774 | 0.8254±0.0907 |
| 364 | Analyte 571 | 27.8454 | 204 | 1.2176±0.0853 | 0.6302±0.0900 | 1.3009±0.1208 | 1.7114±0.2231 |
| 365 | 5-dihydrocortisol 1 | 28.2994 | 255 | 0.0002±0.0000 | 0.0657±0.0102 | 0.0119±0.0122 | 0.0384±0.0207 |
| 366 | 1-Kestose | 30.3273 | 217 | 1.0056±0.0842 | 2.4797±0.4357 | 2.4415±0.2377 | 4.2033±0.4961 |
| 367 | cholestane-3,5,6-triol, (3b,5a,6b-) | 30.5261 | 57 | 2.6207±0.1501 | 0.7057±0.1242 | 3.5097±0.3635 | 3.0567±0.3922 |
| 368 | Analyte 580 | 30.8934 | 218 | 0.3815±0.0262 | 0.1735±0.0902 | 0.2958±0.0324 | 0.5178±0.0625 |
| 369 | Analyte 581 | 31.2097 | 218 | 0.3480±0.0237 | 0.3267±0.0696 | 0.3969±0.0480 | 0.5090±0.0625 |
| 370 | Analyte 583 | 32.8883 | 57 | 1.1168±0.0885 | 0.3817±0.2160 | 2.2694±0.2638 | 1.4721±0.1761 |
| 371 | Analyte 584 | 33.4890 | 73 | 1.7306±0.1108 | 1.8026±0.3056 | 2.9325±0.3128 | 2.5460±0.3528 |

HCK, HT1, HT2, and HT3 stand for flowers in the control, 25 mg kg^-1^ Cd, 50 mg kg^-1^ Cd, and 100 mg kg^-1^ Cd added group (n = 3), respectively (the same below); All data are present as mean ± SE.
