# Supplementary material for: Gas chromatography-time-of-flight mass spectrometry (GC-TOFMS)-based metabonomic response of Salvia miltiorrhiza flowers to cadmium stress
Source: PeerJ. 2026 Apr 27;14:e21149. doi: 10.7717/peerj.21149 (PMC13131354; doi:10.7717/peerj.21149)
Supplement: Supplemental Information 6 [file peerj-14-21149-s006.docx]

Table S4 Differential metabolites in SM flowers with different levels of Cd stress.

| No. | Metabolite | RT (s) |  |  | HT1 |  |  | HT2 |  |  | HT3 |  |
| --- | --- | --- | --- | --- | --- | --- | --- | --- | --- | --- | --- | --- |
|  |  |  | Mass | VIP | *FDR* | Log_2_FC | VIP | *FDR* | Log_2_FC | VIP | *FDR* | Log_2_FC |
| 1 | Oxamic acid | 10.2066,0 | 171 | -- | -- | -- | 1.3979 | 0.0240 | -2.6266 | 1.3681 | 0.0274 | -2.0964 |
| 2 | Ethanolamine | 10.4612,0 | 174 | -- | -- | -- | -- | -- | -- | 1.3633 | 0.0116 | 1.2863 |
| 3 | 2-Deoxyerythritol | 10.8033,0 | 117 | 1.1977 | 0.0428 | 3.0791 | 1.2124 | 0.0263 | 3.3715 | -- | -- | -- |
| 4 | Isoleucine | 10.8212,0 | 158 | -- | -- | -- | -- | -- | -- | 1.3840 | 0.0027 | 1.8596 |
| 5 | Proline | 10.9033,0 | 142 | -- | -- | -- | -- | -- | -- | 1.3836 | 0.0011 | 3.2772 |
| 6 | Glycine 2 | 11.0086,0 | 174 | 1.4533 | 0.0201 | -1.2995 | -- | -- | -- | -- | -- | -- |
| 7 | Succinic acid | 11.1278,0 | 147 | -- | -- | -- | -- | -- | -- | 1.3395 | 0.0214 | 0.6340 |
| 8 | 1-Methylhydantoin 1 | 11.2938,0 | 71 | -- | -- | -- | -- | -- | -- | 1.3562 | 0.0167 | 0.3716 |
| 9 | D-Glyceric acid | 11.3019,0 | 73 | -- | -- | -- | -- | -- | -- | 1.3810 | 0.0050 | 1.0524 |
| 10 | Itaconic acid | 11.4696,0 | 117 | -- | -- | -- | -- | -- | -- | 1.3958 | 0.0011 | 1.3408 |
| 11 | Fumaric acid | 11.6208,0 | 245 | -- | -- | -- | -- | -- | -- | 1.3331 | 0.0297 | 0.6549 |
| 12 | 1-Indanol | 11.6919,0 | 57 | -- | -- | -- | -- | -- | -- | 1.3234 | 0.0319 | 0.2097 |
| 13 | Serine 1 | 11.7178,0 | 204 | -- | -- | -- | -- | -- | -- | 1.3876 | 0.0040 | 0.8522 |
| 14 | Benzyl thiocyanate | 11.8512,0 | 155 | -- | -- | -- | -- | -- | -- | 1.3793 | 0.0040 | 1.4483 |
| 15 | 3-Cyanoalanine | 11.8846,0 | 141 | -- | -- | -- | -- | -- | -- | 1.3852 | 0.0040 | 1.1558 |
| 16 | Threonine 1 | 12.0539,0 | 73 | -- | -- | -- | 1.4640 | 0.0117 | 0.7139 | 1.3923 | 0.0342 | 1.5635 |
| 17 | Glutaric Acid | 12.2209,0 | 147 | -- | -- | -- | -- | -- | -- | 1.3029 | 0.0331 | 0.9714 |
| 18 | Methyl trans-cinnamate | 12.4018,0 | 103 | 1.5055 | 0.0314 | -11.0530 | 1.4789 | 0.0263 | -10.4188 | 1.4004 | 0.0274 | -10.5602 |
| 19 | L-Malic acid | 13.4267,0 | 73 | -- | -- | -- | 1.4335 | 0.0151 | -0.9134 | -- | -- | -- |
| 20 | Asparagine 4 | 13.6229,0 | 115 | 1.4454 | 0.0170 | -1.4811 | 1.4670 | 0.0066 | 1.1107 | 1.3639 | 0.0135 | 0.8978 |
| 21 | 4-Acetamidobutyric acid 2 | 13.6468,0 | 174 | -- | -- | -- | -- | -- | -- | 1.2867 | 0.0457 | 0.4604 |
| 22 | Aspartic acid 1 | 13.7954,0 | 232 | -- | -- | -- | 1.4577 | 0.0137 | 1.0138 | 1.3937 | 0.0015 | 1.4236 |
| 23 | Oxoproline | 13.8593,0 | 156 | -- | -- | -- | 1.4536 | 0.0141 | 0.7275 | 1.3974 | 0.0015 | 1.6879 |
| 24 | 4-Aminobutyric acid 1 | 13.9367,0 | 174 | 1.4842 | 0.0094 | 1.1523 | 1.4572 | 0.0137 | 0.9266 | 1.3881 | 0.0020 | 1.0281 |
| 25 | Maleamate 1 | 14.1668,0 | 55 | 1.4976 | 0.0030 | -1.5232 | 1.4253 | 0.0137 | -0.9041 | 1.3392 | 0.0214 | -0.7209 |
| 26 | Threonic acid | 14.2441,0 | 73 | 1.4948 | 0.0030 | -0.9205 | 1.4753 | 0.0008 | -2.0343 | 1.3829 | 0.0050 | -0.7129 |
| 27 | Phenylethylamine | 14.4078,0 | 174 | 1.5059 | 0.0289 | -12.4852 | 1.4721 | 0.0275 | -2.5126 | -- | -- | -- |
| 28 | α-ketoglutaric acid | 14.4709,0 | 73 | 1.5021 | 0.0247 | -2.0091 | 1.4636 | 0.0059 | -1.0346 | 1.3902 | 0.0046 | -1.0295 |
| 29 | D-Erythronolactone 2 | 14.7215,0 | 117 | 1.4446 | 0.0271 | -0.4395 | 1.4508 | 0.0075 | -1.1224 | -- | -- | -- |
| 30 | Glutamic acid | 14.9878,0 | 246 | 1.4816 | 0.0135 | -0.9716 | -- | -- | -- | 1.2901 | 0.0344 | 0.4138 |
| 31 | Phenylalanine 1 | 15.0975,0 | 218 | 1.4915 | 0.0083 | -1.3329 | -- | -- | -- | 1.3840 | 0.0035 | 1.0842 |
| 32 | Fluorene | 15.1181,0 | 229 | -- | -- | -- | 1.4677 | 0.0066 | 1.8692 | 1.3481 | 0.0124 | 0.9703 |
| 33 | Creatine degr | 15.1769,0 | 73 | 1.4672 | 0.0153 | 0.4673 | 1.4759 | 0.0013 | 1.1518 | 1.3675 | 0.0150 | 0.7674 |
| 34 | Allose 1 | 15.314,0 | 201 | 1.3851 | 0.0397 | 0.5136 | 1.3845 | 0.0240 | 0.5636 | 1.2889 | 0.0319 | 0.5925 |
| 35 | Xylose 1 | 15.3813,0 | 103 | 1.5038 | 0.0002 | 1.1644 | 1.4805 | 0.0002 | 1.5942 | 1.3955 | 0.0015 | 1.5190 |
| 36 | Ribose | 15.4577,0 | 103 | 1.5039 | 0.0002 | 1.3459 | 1.4809 | 0.0002 | 1.4168 | 1.3938 | 0.0016 | 1.2908 |
| 37 | Levoglucosan | 15.9374,0 | 204 | 1.4963 | 0.0019 | 1.0849 | 1.4636 | 0.0102 | 0.9389 | 1.3795 | 0.0161 | 1.0473 |
| 38 | Diglycerol 2 | 16.1528,0 | 205 | 1.4218 | 0.0320 | 0.2515 | -- | -- | -- | -- | -- | -- |
| 39 | 6-Deoxy-D-glucose 2 | 16.2932,0 | 86 | 1.5050 | 0.0133 | 9.9644 | 1.4800 | 0.0137 | 9.1218 | -- | -- | -- |
| 40 | Fucose 2 | 16.3102,0 | 117 | 1.5056 | 0.0196 | 11.7075 | -- | -- | -- | -- | -- | -- |
| 41 | Uracil-5-Carboxylic acid | 16.5636,0 | 357 | 1.4732 | 0.0129 | -1.1657 | -- | -- | -- | -- | -- | -- |
| 42 | 2-Deoxy-D-Glucose 2 | 16.638,0 | 217 | 1.4985 | 0.0013 | 1.2253 | 1.4681 | 0.0059 | 1.0784 | 1.3025 | 0.0396 | 0.4417 |
| 43 | Farnesal 2 | 16.6757,0 | 226 | 1.5035 | 0.0009 | 0.9410 | 1.4662 | 0.0088 | 0.9030 | 1.3920 | 0.0040 | 0.8521 |
| 44 | α-D-Glucosamine 1-Phosphate | 17.1732,0 | 103 | 1.2467 | 0.0012 | -5.8085 | 1.2451 | 0.0012 | -5.8761 | 1.4010 | 0.0165 | -16.4835 |
| 45 | Citric acid | 17.1966,0 | 273 | 1.5044 | 0.0001 | -2.7897 | 1.4789 | 0.0002 | -2.2418 | 1.3997 | 0.0004 | -2.1342 |
| 46 | Mannose 2 | 18.0639,0 | 409 | -- | -- | -- | 1.4526 | 0.0137 | -1.5678 | -- | -- | -- |
| 47 | D-Galacturonic acid 1 | 18.4104,0 | 333 | -- | -- | -- | 1.4276 | 0.0270 | 1.4272 | -- | -- | -- |
| 48 | Tyrosine 1 | 18.4955,0 | 218 | -- | -- | -- | 1.4254 | 0.0206 | 1.0009 | 1.3487 | 0.0351 | 1.5572 |
| 49 | Guanine 2 | 19.0753,0 | 324 | -- | -- | -- | 1.4801 | 0.0443 | 10.8251 | -- | -- | -- |
| 50 | 4-Hydroxymethyl-3-methoxyphenoxyacetic acid | 19.3326,0 | 324 | 1.2202 | 0.0392 | 3.3894 | 1.2681 | 0.0125 | 4.4229 | 1.1614 | 0.0274 | 3.9974 |
| 51 | Myo-inositol | 19.9229,0 | 217 | -- | -- | -- | 1.4053 | 0.0413 | 1.1493 | -- | -- | -- |
| 52 | d-Glucoheptose 1 | 20.3025,0 | 319 |  |  |  | 1.4506 | 0.0175 | 1.6524 | -- | -- | -- |
| 53 | β-Mannosylglycerate 1 | 20.9344,0 | 217 | 1.4264 | 0.0499 | 1.4923 | 1.4500 | 0.0132 | 1.6125 | -- | -- | -- |
| 54 | Fructose 2,6-biphosphate degr prod 2 | 20.9696,0 | 211 | -- | -- | -- | 1.4252 | 0.0166 | -0.7794 | 1.2667 | 0.0360 | -0.8084 |
| 55 | Glucose-6-phosphate 1 | 21.8749,0 | 387 | 1.4582 | 0.0185 | -1.2521 | -- | -- | -- | 1.2898 | 0.0378 | -0.8027 |
| 56 | Purine riboside | 22.3357,0 | 204 | -- | -- | -- | -- | -- | -- | 1.3584 | 0.0319 | 1.8197 |
| 57 | DL-dihydrosphingosine 1 | 23.3274,0 | 204 | -- | -- | -- | 1.4146 | 0.0383 | 1.2783 | -- | -- | -- |
| 58 | Lactose 2 | 24.7758,0 | 307 | -- | -- | -- | 1.4492 | 0.0166 | 1.9267 | 1.3568 | 0.0245 | 1.8986 |
| 59 | Maltose | 25.2801,0 | 160 | -- | -- | -- | 1.3997 | 0.0336 | 1.5093 | 1.3282 | 0.0380 | 1.5923 |
| 60 | Lactobionic acid 1 | 25.3577,0 | 193 | 1.4008 | 0.0250 | -1.9713 | -- | -- | -- | 1.3336 | 0.0396 | 1.3208 |
| 61 | Melibiose 1 | 26.0543,0 | 204 | -- | -- | -- | -- | -- | -- | 1.3349 | 0.0462 | 1.3290 |
| 62 | Galactinol 1 | 26.892,0 | 204 | -- | -- | -- | -- | -- | -- | 1.3662 | 0.0274 | 2.5863 |
| 63 | Chlorogenic acid 1 | 27.7715,0 | 219 | 1.4392 | 0.0297 | -0.8938 | -- | -- | -- | -- | -- | -- |
| 64 | 1-Kestose | 30.3273,0 | 217 | -- | -- | -- | 1.4181 | 0.0263 | 1.2797 | 1.3704 | 0.0217 | 2.0635 |
| 65 | Phenylacetaldehyde 2 | 9.39038,0 | 91 | 1.4705 | 0.0097 | -1.8929 | -- | -- | -- | 1.2988 | 0.0453 | 1.7085 |
| 66 | Valine | 9.73813,0 | 144 | -- | -- | -- | -- | -- | -- | 1.3651 | 0.0150 | 2.1827 |

The differential metabolites were selected based on VIP value > 1 and *FDR* (*p* value adjusted using False Discovery Rate) < 0.05; FC, ratio of metabolite content between Cd stressed groups (HT1, HT2, and HT3) and control group (HCK).
